# Effect of Acupuncture on Movement Function in Patients with Parkinson’s Disease: Network Meta-Analysis of Randomized Controlled Trials

**DOI:** 10.3390/healthcare9111502

**Published:** 2021-11-05

**Authors:** Miri Kwon, Moon Joo Cheong, Jungtae Leem, Tae-hun Kim

**Affiliations:** 1 Department of Clinical Korean Medicine, College of Korean Medicine, Graduate School, Kyung Hee University, Seoul 02447, Korea; dove58@naver.com; 2 Rare Diseases Integrative Treatment Research Institute, Wonkwang University, Jangheung Integrative Medical Hospital, Iksan 59338, Korea; sasayayoou@naver.com; 3 Research Center of Traditional Korean Medicine, Wonkwang University, Iksan 54538, Korea; 4 Korean Medicine Clinical Trial Center, Korean Medicine Hospital, Kyung Hee University Medical Center, Seoul 02447, Korea

**Keywords:** network meta-analysis, meta-analysis, Parkinson’s disease, motor symptom, systematic review, acupuncture

## Abstract

We aimed to compare the effectiveness of some different acupuncture modalities on motor function using the unified Parkinson disease rating scale (UPDRS)-III scores of idiopathic Parkinson’s disease (PD) via pairwise and network meta-analyses (NMA) of randomized controlled trials (RCTs). The Cochrane risk of bias assessment tool was used to assess the methodological quality of the included RCTs. A frequentist approach-based random effect model NMA was performed. Seventeen RCTs with 1071 participants were included. The five following modalities were identified: combination of conventional medication (levodopa) with (1) electroacupuncture (ELEC), (2) manual acupuncture (MANU), (3) bee venom acupuncture (BEEV), (4) sham acupuncture (SHAM), and (5) conventional medication alone (CONV). In NMA on UPDRS-III, BEEV was the best modality compared to CONV (mean difference [MD]) −7.37, 95% confidence interval [−11.97, −2.77]). The comparative ranking assessed through NMA was suggested to be BEEV, MANU, ELEC, SHAM, and CONV. Regarding daily activity assessment (UPDRS-II), the magnitude of effectiveness was in the order of BEEV, ELEC, MANU, SHAM, and CONV. Combination treatment with BEEV (MANU or ELEC) and CONV can be recommended to improve motor function in PD patients. Due to the limited number of included RCTs, further NMA with more rigorous RCTs are warranted.

## 1. Introduction

Parkinson’s disease (PD) is a degenerative neurological disorder associated with dopaminergic cell loss in the substantia nigra and other brain structures characterized by several movement symptoms, such as tremor, rigidity, tremor at rest, and postural instability [1]. PD is the second most common neurodegenerative disorder after Alzheimer’s dementia. The prevalence of PD is increasing faster than in other neurological diseases [1,2]. The prevalence increases with age, and in most cases, the cause is unknown [3,4]. Approximately 6.1 million people worldwide were diagnosed with PD in 2016, which is more than double that of 1990 [5]. The movement symptoms of PD are managed using a combination of conventional medications, such as levodopa, carbidopa, dopamine agonists, and monoamine oxidase B inhibitors [5]. On the other hand, if levodopa is administered for a long period, treatment may not be continued due to side effects, such as the on-off phenomenon [6]. Levodopa-induced dyskinesia also impairs the quality of life of patients with PD, making effective treatment difficult [7]. In a previous study, more than 40% of patients with PD experienced wear-off and levodopa-induced dyskinesia, which lowered drug adherence [8]. If conventional drugs are ineffective, several surgical strategies, such as deep brain stimulation (DBS) or radiofrequency ablation, could be considered [9]. However, there are a number of complications associated with a surgical approach, and patient expectations after surgery are sometimes not fulfilled [10]. In addition, as the disease progresses, the burden on caregivers increases because of frequent nursing home visits, longer hospital stays, and higher rates of emergency room visitation [11]. Therefore, in addition to conventional management, alternative therapeutic options are needed to manage various symptoms considering the characteristics of PD, which has a long disease duration.

Recently, various complementary and integrative medicine (CIM) therapies, such as acupuncture, herbal medicine, qi-gong, massage, yoga, meditation, and music therapy, have been widely utilized in clinical practice for PD symptom management [12]. Acupuncture is one of the most commonly used CIM interventions for the management of patients with PD [13]. In previous studies, acupuncture improved motor symptoms, non-motor symptoms, quality of life, and disease progression, and decreased the adverse events and dosage of anti-parkinsonian medication [13]. Several clinical studies and systematic reviews have shown the effects of various types of acupuncture treatment combined with conventional medication on motor symptom improvement using the unified PD rating scale (UPDRS) [14,15,16,17].

However, it is unknown which type of acupuncture treatment is the preferred option because the acupuncture treatment applied in each study is different. Most clinical research and systematic reviews have compared two interventions at a time. Multiple intervention comparison research designs are not common. However, in clinical practice, physicians are curious about which treatment is more effective among the various widely used treatments. However, in terms of time and cost, it is difficult to conduct direct comparative (head-to-head) studies on various acupuncture treatments, and the need to compare multiple interventions at a time is increasing. Nowadays, a novel methodology named ‘network meta-analysis (NMA)’ is used to simultaneously estimate the relative effect of various interventions [18,19]. NMA results help stakeholders to make decisions by providing a combined quantitative effect size acquired from direct and indirect comparisons of different interventions [20].

This study aimed to compare the effect on movement symptom improvement in patients with PD about several acupuncture types combined with conventional medication (CM), such as manual acupuncture (MA), electroacupuncture (EA), and bee venom acupuncture (BVA), compared with placebo acupuncture or conventional medication only. We adopted conventional systematic review, pairwise meta-analysis (PMA), and NMA methodology to compare the effect size of various acupuncture types to help with decision making regarding the management of patients with PD.

## 2. Materials and Methods

We followed the preferred reporting items for systematic reviews and meta-analyses for network meta-analysis checklist (PRISMA-NMA) [21]. This review protocol was registered with the Open Science Framework on 7 August 2021 (https://osf.io/q8n7z/).

### 2.1. Search Strategy

Eligible studies were systematically searched from their inception to June 2021 using Medline (via PubMed), Cochrane Library, Embase (via Elsevier), China National Knowledge Infrastructure, Korea Citation Index (KCI), NDSL, Research Information Sharing Service, and Oriental Medicine Advanced Searching Integrated System. A mixture of free words and medical subject headings were used for PD and acupuncture. There were no language restrictions. The search strategy in Medline (via PubMed) is as follows:

#1 parkinson disease [Mesh] OR parkinson disease OR Idiopathic Parkinson’s Disease OR Lewy Body Parkinson’s Disease OR Parkinson’s Disease, Idiopathic OR Parkinson’s Disease, Lewy Body OR Parkinson Disease, Idiopathic OR Parkinson’s Disease OR Idiopathic Parkinson Disease OR Lewy Body Parkinson Disease OR Primary Parkinsonism OR Parkinsonism, Primary OR Paralysis Agitans.

#2 acupuncture [Mesh] OR Acupuncture OR Pharmacopuncture OR Acupuncture Treatment OR Acupuncture Treatments OR Treatment, Acupuncture OR Therapy, Acupuncture OR Pharmacoacupuncture Treatment OR Treatment, Pharmacoacupuncture OR Pharmacoacupuncture Therapy OR Therapy, Pharmacoacupuncture OR Acupotomy OR Acupotomies OR Electroacupuncture OR Bee Venoms OR Venoms, Bee OR Bee Venom OR Venom, Bee OR Apis Venoms OR Venoms, Apis OR Apitoxin OR Honeybee Venoms OR Venoms, Honeybee OR Honeybee Venom OR Venom, Honeybee OR Fire needle therapy OR Fire acupuncture.

#3 #1 AND #2: A detailed explanation of the search terms used in each database is provided in Supplementary Materials Digital Content 1.

### 2.2. Eligible Criteria

#### 2.2.1. Type of Studies

Only randomized controlled clinical trials (RCTs) were included. We did not include cluster randomized clinical trials. Other study designs, such as animal studies, uncontrolled tests, or case reports, were excluded. Multi-armed trials (≥ three arms) were included if they did not violate the eligibility criteria.

#### 2.2.2. Type of Participants

Patients diagnosed with idiopathic PD were included without limitation of age, sex, race, severity, or duration of disease. Patients other than those with idiopathic PD, such as Parkinson’s syndrome, were excluded.

#### 2.2.3. Type of Intervention Used in the Experimental and Control Groups

The experimental group intervention consisted of different types of acupuncture treatment combined with CM. In the control group, we selected L-dopa, which has been an effective gold standard dopamine-based medication for movement symptom management for approximately 60 years, as an essential medication for the control group (CM) [22,23]. Studies were included if the combination of L-dopa and other drugs was equally applied to the acupuncture and control groups. However, studies in which treatment medication therapy was performed only with other drugs without L-dopa were excluded. Acupuncture treatments included electroacupuncture (EA), MA, or BVA. We excluded combined acupuncture treatments, such as EA + BVA or MA + BVA, to evaluate the therapeutic effect of each acupuncture intervention type. The intervention in the control group was defined as CM therapy alone or CM + sham acupuncture treatment. We did not restrict the duration, dosage, or frequency of treatment.

#### 2.2.4. Type of Outcome Measure

The primary outcome of our study was the motor function of patients with PD evaluated using the UPDRS-III scale [24]. The secondary outcomes were daily life activity scores using the UPDRS-II [24]. The Movement Disorder Society UPDRS (MDS-UPDRS) was excluded because it is different from UPDRS [25]. The timing of the outcome assessment was selected immediately after the end of the acupuncture treatment session. Data acquired during the follow-up assessment were not considered.

### 2.3. Study Selection and Data Extraction

Two reviewers (M.K. and J.L.) independently conducted the study selection and data extraction.

Disagreement between the two researchers was resolved by discussion with a third independent reviewer (M.J.). Duplicate publications, patients diagnosed with Parkinsonism syndrome, and cases in combination with other treatments were excluded. A standardized data collection form developed during the pilot process using Excel was utilized during the data extraction process. The extracted items were as follows: sample size and the number of dropouts, first author, year of publication, location, age, sex, disease severity, disease duration, treatment intervention, control group intervention, treatment period, and outcome variables. We contacted the corresponding author to acquire sufficient data if there was insufficient information in the published article via e-mail. EndNote X9 (EndNote version X9, Thomson Reuters, CA, USA) was used for article selection and management.

### 2.4. Risk of Bias Assessment

Two independent researchers (J.L., M.K.) used the Cochrane risk of bias assessment tool to evaluate the quality of the research methods of the included studies [26]. Random sequence generation; allocation concealment; blinding of participants, personnel, and outcome assessors; incomplete outcome data; selective outcome reporting; and other sources of bias were graded as low, unclear, and high. Disagreement between the two researchers was resolved by discussion with a third independent reviewer (M.J.). Review Manager (RevMan) version 5.4 software was used to illustrate the risk of bias.

### 2.5. PMA

In the PMA, we conducted a conventional direct comparison of the two study arms. Data synthesis was performed using the Review Manager (RevMan) ([Computer program]. Version 5.4, The Cochrane Collaboration, 2020). The random effect model was adopted because it was judged that there was heterogeneity due to differences in the study design, such as baseline characteristics, number of interventions, and methods among the included studies. The mean difference (MD) for the continuous variables and 95% confidence interval (CI) were used to assess the effect size of the intervention on UPDRS-III and II. Heterogeneity was determined by both the chi-square (χ2) test and Higgins’ $I^{2}$ statistic. The heterogeneity interpretation based on the $I^{2}$ statistic is considered not to be important (0 to 40%), moderate heterogeneity (30% to 60%), substantial heterogeneity (50% to 90%), and considerable heterogeneity (75% to 100%) [27]. A *p*-value of ≤0.1 was considered to indicate significant heterogeneity [28].

### 2.6. NMA

#### 2.6.1. Assumptions of the NMA

The frequentist model was utilized for the NMA, combining direct and indirect evidence using R version 4.1.0 (A language and environment for statistical computing. R Foundation for Statistical Computing, Vienna, Austria)) using the Netmeta package [29]. There are several assumptions for NMA, such as connectivity, homogeneity, transitivity, and consistency [30]. Connectivity was visually verified by connecting each network node with a line using a network plot. Homogeneity was assessed using the Cochrane Q statistic or the I^2^ score. In our study, a random effect model was applied, as it was judged that there was heterogeneity between studies due to differences in study design or interventions [30,31]. When evaluating transitivity, it is necessary to explore the distribution of effect modifiers and determine their effects on the effect size. In our study, we qualitatively compared the sample size, age, sex, disease duration, severity, treatment dosage, and period for transitivity assessment [30]. Consistency is a quantitative statistical evaluation of transitivity. Consistency was statistically evaluated using the net-splitting method [32].

#### 2.6.2. Statistical Assessment

The network forest plot presented with MD and 95% CI of each intervention was used to rank each treatment strategy for visual and statistical verification. The P-score was also used to rank treatment, which assesses certainty that a specific intervention is better than competing inventions. The P-score is nearly identical to the numerical values of SUCRA in the Bayesian model NMA [33]. For the consistency assumption, we checked both global (network level) and local approaches (particular contrast of intervention level) [21]. In the global approach, we used the ‘decomp.design’ function of R software to assess consistency under the assumption of a full design-by-treatment interaction random effect model [34]. Q statistics were used to assess inconsistency in the global approach. If the *p*-value for the Q statistics was below 0.05, it was assumed that significant inconsistency (disagreement) existed in the global network. In the local approach, we adopted the net-splitting method to split the network estimation of the effect size on each intervention into direct and indirect evidence using the Facenetsplit function of R software. It calculates the difference between direct and indirect estimates and assesses whether the difference is statistically significant [34]. Net-split plots were also provided for visual inspection of inconsistencies between direct and indirect comparisons. If the *p*-value for the net-split analysis was below 0.05, it was assumed that significant inconsistency (disagreement) existed in a specific local loop, which indicates a considerable difference between indirect and direct effect size estimation. If there were significant disagreements in the local or global approach, we conducted a sensitivity analysis by sequentially excluding studies one by one. If we identified which studies were inconsistent, we excluded studies from the NMA. A net league table is also presented. The upper right triangle presents the effect size estimated by only direct comparison, which is similar to the pairwise comparison. As direct comparison does not exist in all treatment comparisons, there are several blanks in the upper triangle. The lower left triangle provides a pooled estimation of the direct and indirect comparisons of the effect size.

#### 2.6.3. Sensitivity Analysis

NMA was performed by sequentially removing each study one by one to confirm whether a specific study excessively affected the overall result. The results were visually and statistically checked to determine whether the results were consistent with the overall trend.

### 2.7. Publication Bias

We used a conventional funnel plot for visual inspection of the publication bias. We also used Egger’s test to statistically assess publication bias [35]. If the *p*-value for Egger’s test was greater than 0.05, it indicated no evidence of publication bias.

## 3. Results

### 3.1. Characteristics of the Included Studies and Network Geometry

**Figure 1:**
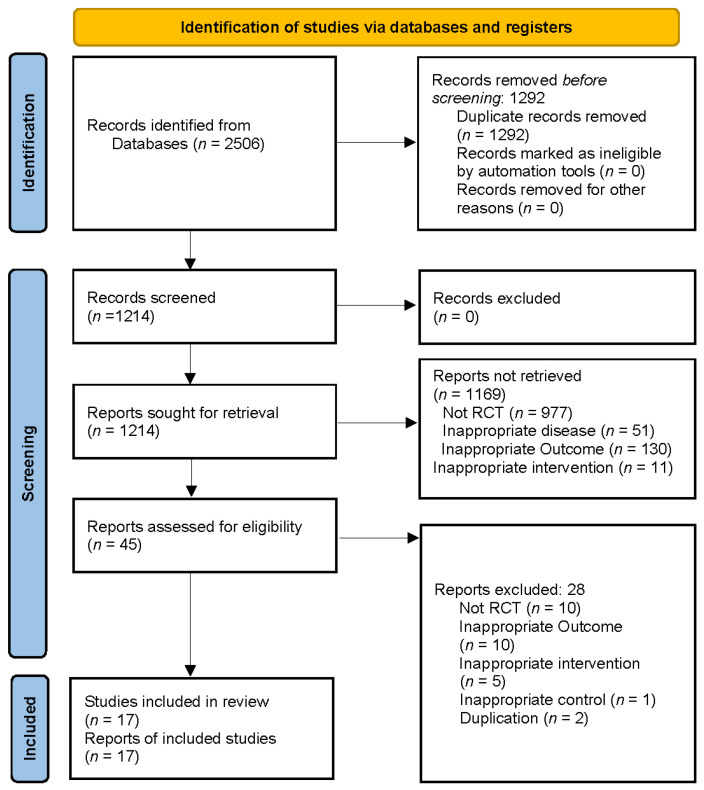
PRISMA flow diagram.

A total of 2505 articles were screened from eight databases. After careful review of the title and abstract, 17 articles were finally included (Figure 1). In 17 RCTs, 1071 participants were included. A list of the 28 studies excluded after reviewing the full text is provided in Supplementary Materials Digital Content 2. Eight articles were written in English [36,37,38,39,40,41,42,43], one article was written in Japanese [44], two in Korean [15,45], and six articles were written in Chinese [14,46,47,48,49,50]. Detailed characteristics of the included studies including publication year, first author, country, sample size (initial and final), age, sex, disease severity, disease duration, CM dosage (mg/day), treatment and control group intervention, and treatment period are described in Table 1.

Five types of arms were identified: (1) manual acupuncture + conventional drug (MANU), (2) electroacupuncture + conventional drug (ELEC), (3) BVA + conventional drug (BEEV), (4) sham acupuncture + conventional drug (SHAM), and (5) conventional drug alone (CONV). Fifteen RCTs had two armed designs, and only two RCTs had three armed designs (one RCT included MANU vs. SHAM vs. CONV [38], and one RCT included BEEV vs. MANU vs. CONV [41]). Seven trials included the ELEC arm, nine trials included the MANU arm, and two included the BEEV arm. Therefore, a total of 18 comparisons (36 treatment arms) were included in the 17 RCTs. Six RCTs compared ELEC vs. CONV [14,40,42,46,48,49]; five RCTs compared MANU vs. CONV [38,41,44,47,50]; one RCT compared ELEV vs. SHAM [36]; five RCTs compared MANU vs. SHAM [15,37,38,43,45]; and one RCT compared BEEV vs. SHAM [39]. Detailed descriptions of each intervention, including the acupuncture point, needle stimulation, retention time, treatment duration, and frequency, are described in Table 2.

### 3.2. Risk of Bias of the Included Studies

In random sequence generation, five studies were graded as unclear [36,40,46,48,49]. In allocation concealment, 10 studies were graded as unclear [14,15,36,40,44,45,46,48,49,50]. Nine studies were graded as high in blinding of participants because several articles were add-on study designs that cannot blind participants [14,38,41,42,43,46,48,49,50]. Furthermore, four studies were graded as unclear. In the blinding of outcome assessment, 11 studies were graded as unclear [14,15,39,43,44,45,46,47,48,49,50]. In incomplete outcome data, two studies were graded as unclear [15,44]. Six studies were graded as high as the DR was more than 10% [36,37,38,39,41,45]. One study was graded as high in selective reporting because it did not report the outcome variables previously described in the protocol [36]. Detailed visualization of each study with the risk of bias graph is presented in Supplementary Materials Digital Content 3.

### 3.3. Primary Outcome (Movement Function, UPDRS-III): PMA

In PMA of movement function evaluated by UPDRS-III, statistical significance was shown between the following comparisons presented with MD and 95% CI **(in favor of bold marks):** (1) **electroacupuncture + CM (ELEC)** vs. CM (CONV) (MD −3.63, 95% CI −6.05 to −1.21); (2) **manual acupuncture + CM (MANU)** vs. CONV (MD −3.90, 95% CI −6.24 to −1.57); (3) **electroacupuncture + CM (ELEC)** vs. sham acupuncture + CM (SHAM) (MD −18.10, 95% CI −30.31 to −5.89). In other comparisons, such as BVA + CM (BEEV) vs. CONV, MANU vs. SHAM, and BEEV vs. SHAM, the acupuncture modality tended to be more effective than the control group, but the difference was not statistically significant. Detailed descriptions of the effect size and each trial-based forest plot are provided in Supplementary Materials Digital Content 4.

### 3.4. Secondary Outcome (Daily Life Activity, UPDRS-II): PMA

In PMA of daily life activity evaluated by UPDRS-II, statistical significance was shown between the following comparisons presented with MD and 95% CI **(in favor of bold marks):** (1) **ELEC** vs. CONV (MD −4.50, 95% CI −6.19 to −2.80); (2) **MANU** vs. CONV (MD −4.07, 95% CI −4.87 to −3.27). In other comparisons, such as BEEV vs. CONV, ELEC vs. SHAM, and MANU vs. SHAM, the acupuncture modality showed a tendency to be more effective than the control group, but this was not statistically significant. Detailed descriptions of the effect size and each trial-based forest plot are provided in Supplementary Materials Digital Content 4.

### 3.5. Primary Outcome (Movement Function, UPDRS-III): NMA

#### 3.5.1. Assumption of NMA and Network Geometry

**Figure 2:**
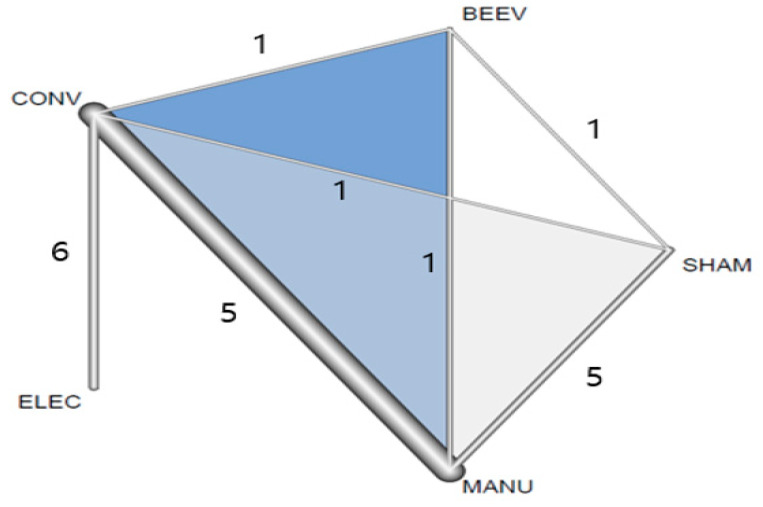
Network geometry of the included studies on UPDRS-III (Net graph). BEEV, bee venom acupuncture + conventional drug therapy; CONV, single conventional drug therapy; ELEC, electroacupuncture + conventional drug therapy; MANU, manual acupuncture + conventional drug therapy; SHAM, sham acupuncture + conventional drug therapy; UPDRS, Unified PD rating scale.

As explained in the Methods section, we decided to adopt a random effect model in the homogeneity assumption. In the transitivity assumption, the research team agreed on the transitivity of the included studies using Table 1 and Table 2. We assessed the consistency assumption using a global and local approach. In the global approach, we found significant inconsistencies (*p* < 0.05). In the local approach, we found inconsistency due to a study that compared ELEC and CONV (Lei 2016 [36]). After we excluded the study (Lei 2016 [36]) according to the study protocol, the consistency assumption was satisfied at the local and global levels. Net-split graphs that include direct estimates, indirect estimates, and network estimates for consistency assessment are provided in Supplementary Materials Digital Content 5. The connectivity assumption was confirmed through network geometry (net graph), which is a visual presentation of the links in the included studies (Figure 2). After excluding the study (Lei 2016 [36]), in the network analysis of the primary outcome, there were five nodes (ELEC, BEEV, MANU, CONV, SHMA) from 16 studies and 20 pairwise comparisons from seven types of comparison pairs (edges). The number of included comparisons in each edge is shown in Figure 2.

#### 3.5.2. Comparative Effectiveness of the Acupuncture Modality in UPDRS-III

**Figure 3:**
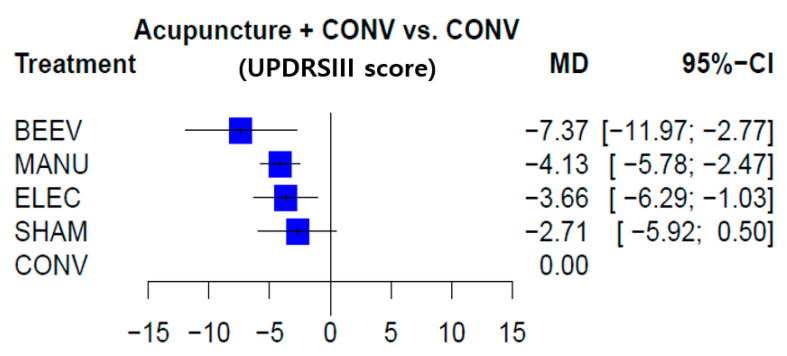
Treatment level network meta-analysis forest plot (UPDRS-III). BEEV, bee venom acupuncture + conventional drug therapy; CONV, single conventional drug therapy; ELEC, electroacupuncture + conventional drug therapy; MANU, manual acupuncture + conventional drug therapy; SHAM, sham acupuncture + conventional drug therapy; UPDRS, Unified PD rating scale.

The probabilities of treatment ranking (P-score) among the included interventions were as follows: BEEV (0.9509), MANU (0.6325), ELEC (0.5349), SHAM (0.3685), and CONV (0.0132). According to the P-score, BEEV is most likely the best acupuncture modality for movement function assessed by the UPDRS-III (Figure 3 and Table 3). Mixed effect estimates (combining direct and indirect estimates) for each intervention compared with CONV were as follows **(in favor of bold marks)**: **BEEV** (MD −7.37, 95% CI −11.97 to −2.77); **MANU** (MD −4.13, 95% CI −5.78 to −2.47); **ELEC** (MD −3.66, 95% CI −6.29 to −1.03); SHAM (MD −2.71, 95% CI −5.92 to 0.50). BEEV, MANU, and ELEC were superior to CONV in UPDRS-III. However, SHAM was not statistically significant. No difference was observed in the comparison between the different acupuncture modalities (Table 3).

The part highlighted in BOLD with underlining is a comparison with statistically significant results. The upper right triangle presents the effect size estimated using only direct comparison. As direct comparison does not exist in all treatment comparisons, there are several blanks in the upper right triangle. The lower left triangle provides a pooled estimation of the direct and indirect comparisons of the effect size.

#### 3.5.3. Sensitivity Analysis

After excluding one study in the sensitivity analysis, (1) BEEV showed a tendency to be most effective in all 16 analyses; (2) in three sensitivity analyses (when excluding [40,44,47]), the ranking between MANU and ELEC was changed, with ELEC showing a better effect; and (3) CONV tended to have the smallest effect size throughout the analysis (Supplementary Materials Digital Content 6).

### 3.6. Secondary Outcome (Daily Life Activity, UPDRS-II): NMA

#### 3.6.1. Assumption of NMA and Network Geometry

Homogeneity and transitivity assumptions are the same as those described in Section 3.5.1. We assessed the consistency assumption via a global and local approach and found no evidence of inconsistency after excluding the study by Lei [36]. The connectivity assumption was confirmed through network geometry (Supplementary Materials Digital Content 7). There were five nodes (ELEC, BEEV, MANU, CONV, and SHMA) from 10 studies and 14 pairwise comparisons from six types of comparison pairs (edges).

#### 3.6.2. Comparative Effectiveness of the Acupuncture Modality in UPDRS-II

The probability of treatment as the best treatment option was presented through a measure called the P-score. The P-scores of the included modalities were as follows: BEEV (0.8971), ELEC (0.6685), MANU (0.5527), SHAM (0.3801), and CONV (0.0016). According to the P-score, BEEV was found to most likely be the best acupuncture modality for activities of daily life assessed by the UPDRS-II. The estimated effect size of each acupuncture modality compared to CONV via the NMA is presented in a treatment level forest plot and league table (Supplementary Materials Digital Content 7). In the treatment level forest plot and league table, the network estimate of the effect size (combining direct and indirect estimates) compared to CONV was as follows **(in favor of bold marks)**: **BEEV** (MD −6.07, 95% CI −9.41 to −2.72); **ELEC** (MD −4.50, 95% CI −6.19 to −2.80); **MANU** (MD −4.08, 95% CI −4.84 to −3.32); **SHAM** (MD −3.21, 95% CI −5.72 to −0.70). BEEV, MANU, ELEC, and SHAM were superior to CONV in the UPDRS-II. As UPDRS-II is a secondary outcome, we did not conduct an additional sensitivity analysis.

### 3.7. Adverse Events (AEs)

AEs were also assessed in the present study. Based on the comparisons, AE rates are summarized as follows. Reported AEs according to RCT design are as follows: (1) ELEC vs. CONV design: 3/30 AEs in the ELEC group vs. 12/30 AEs in the CONV group were reported (Chen 2012 [14]); (2) MANU vs. CONV: not reported; (3) BEEV vs. CONV: 1/18 AEs in the BEEV group were reported (Cho 2012 [41]); (4) ELEC vs. SHAM: not reported; (5) MANU vs. SHAM: 1/47 in the MANU group was reported (Kluger 2016 [43]); and (6) BEEV vs. SHAM: 4/20 AEs in the BEEV group were reported (Hartmann 2016 [39]).

### 3.8. Publication Bias

**Figure 4:**
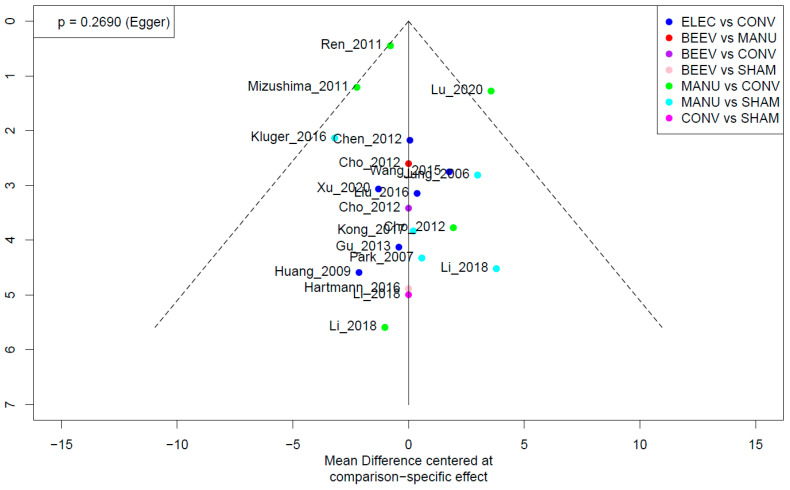
Network funnel plot: UPDRS-III score.

A network funnel plot of the primary outcome (UPDRS-III) was constructed. There was no significant asymmetry seen in the visual inspection of the funnel plot (Figure 4). The Egger’s test did not find any significant evidence of publication bias (*p* = 0.269). In the secondary outcome (UPDRS-II), there was no evidence of publication bias (Supplementary Materials Digital Content 7).

## 4. Discussion

### 4.1. Summary of Findings

The purpose of this PMA and NMA was to explore which acupuncture treatment modality combined with conventional drug therapy is more effective than conventional drug therapy alone for the improvement of motor symptoms (UPDRS-III) and activity of daily living (UPDRS-II) in PD. In NMA on motor symptoms (UPDRS-III), the order of effect size was BEEV, MANU, ELEC, SHAM, and CONV. BVA combination therapy is most likely the best modality for movement symptoms. In NMA on activities of daily living (UPDRS-II), the order of the effect size was BEEV, ELEC, MANU, SHAM, and CONV. BVA combination therapy is most likely to be the best modality for activities of daily living. No serious AEs were observed.

### 4.2. Implications for Clinical Practice and Suggestions for Further Research

The mechanism and therapeutic effect of acupuncture on PD have been elucidated in several studies. In a PD animal model, the expression of tropomyosin receptor kinase B (trkB) was increased in the ipsilateral substantia nigra, and a neuroprotective effect on neuronal cell death was revealed [51]. It also exhibits dopaminergic neuroprotective effects by inducing hypothalamic melanin-concentrating hormone biosynthesis [52]. As a result, it is possible to improve motor behavior while reducing the loss of dopaminergic neurons [51]. In a mechanistic study with functional MRI, acupuncture treatment for patients with PD demonstrated that the putamen and primary motor cortex were activated, and motor function was improved [51]. The mechanism of BVA has also been studied. Apamin toxin contained in BEEV is a polypeptide neurotoxin that blocks Ca2+ activated K + (SK) channels and induces hyperpolarization of dopaminergic neurons, thereby partially rescuing dopaminergic neurons in dissociated midbrain cell cultures [53]. BVA increases the size and number of neurons and striatal dopamine and protects dopaminergic neurons. Therefore, when BEEV is used alone or in combination with conventional drugs for PD, neuronal degeneration is alleviated, and movement disorders are reduced [54]. Several systematic reviews and meta-analyses of RCTs have also been published about the effect and safety of several acupuncture treatment modalities on PD [13,16,17,55].

However, it is unclear which acupuncture modality has a better effect and should be considered in clinical practice and research on PD. Therefore, we performed this NMA to help clinicians and researchers decide which acupuncture modality to use for PD. Although several NMA studies on acupuncture for various diseases have been reported [56,57,58], this is the first NMA study of acupuncture on PD. In our study, BEEV seems to be the best therapeutic option for motor symptoms and activities of daily living in patients with PD. However, the 95% CI overlapped different acupuncture modalities. Therefore, caution should be exercised when applying the results of this study to clinical practice and clinical research. In terms of effect size, the minimal clinical important differences (MCIDs) of the UPDRS motor scores were 2.5 points (minimal effect), 5.2 points (moderate effect), and 10.8 points (large effect) [59]. It was similar (approximately 5–7) in other MCID studies on the UPDRS III scores in patients with PD [60,61,62]. Considering the previous results of the MCID study, our results for the BEEV group showed a clinically significant moderate effect. The effect sizes of ELEC and MANU existed between minimal and moderate effects.

From a clinical perspective, even though BEEV might be the best option for motor symptoms and activities of daily living, MANU/ELEC might be an appropriate option for several motor symptoms [63]. In the presence of severe tremors, it may be difficult to use ELECs in the distal extremities. Therefore, physicians can try electroacupuncture treatment using acupuncture points on the scalp. BEEV might be inappropriate in some cases due to the risk of AEs, such as anaphylaxis [64]. In our results, MANU and ELEC had the best effect after BEEV in UPDRS-II and III. Therefore, if it is difficult to apply BEEV due to Aes, MANU or ELEC could be used as an alternative approach. However, the superiority between MANU and ELEC could not be determined in our study. In the sensitivity analysis, after excluding a long-term follow-up manual acupuncture study [44], ELEC was found to be better than MANU in UPDRS-III. Therefore, it might be possible that the treatment dose (number of sessions) might be an important factor for the therapeutic effect, but as the number of RCTs included in this study was relatively small, we could not conduct further analysis. As head-to-head comparison studies on ELEC and MANU are not common, meta-regression analysis or real-world evidence-based research with health insurance data are needed to address this issue. In summary, when deciding on the acupuncture treatment strategy for patients with PD in clinical practice, we need to consider several factors, such as applicability, adherence, AEs, and target symptoms. In real-world clinical practice, as an overlap of 95% CI of the effect size is clearly visible, it is recommended that BEEV combined with MA with/without electrical stimulation is recommended. Based on the results of this study, in clinical practice, we recommend using electroacupuncture on GB20 (Fengchi) and GB34 (Yanglingquan) for approximately 20–30 min in patients with PD from a clinical point of view. Since bee venom is a natural toxin, in terms of safety, therapeutic dosage is very important. In our study, the total amount of BEEV per session and total number of treatment sessions applied in our review were 100 μg (in 1 mL of NaCl 0.9%) for 11 sessions [39] and 50 μg (in 1 mL of NaCl 0.9%) for 16 sessions [41], respectively. With regard to safety, attention should be paid to side effects (such as anaphylaxis) when higher doses of BEEV than those reported in this study are applied. In addition to predictable dose-dependent side effects, non-predictable side effects due to individual sensitivity should also be considered.

Interestingly, the combined treatment of sham acupuncture with conventional medicine group (SHAM) was superior to the conventional medicine alone group (CONV). Placebo acupuncture is known to have a larger non-specific effect than other physical and pharmacological placebo modalities [65]. Sham acupuncture is known to be more effective than usual care or wait-list control groups for musculoskeletal diseases, such as non-specific low back pain [66]. Our study suggests that sham acupuncture might also have considerable non-specific effects on degenerative neurological diseases, such as PD. Therefore, a sham acupuncture-controlled design might underestimate the effect of acupuncture treatment. A pragmatic clinical study on comprehensive acupuncture treatment (combining ELEC, MANU, and BEEV) compared to an active control group (such as rehabilitation, medication, qi-gong) might be a more appropriate design to address physicians’ questions about which intervention should be added to CM.

### 4.3. Strengths and Limitations

Our study had several strengths. This is the first NMA acupuncture study for PD in an area that is difficult to conduct clinical trials due to resource limitations and research priorities. We included studies across multiple databases without language restrictions. The assumptions for performing the network meta-analysis were systemically reviewed, and there was a methodological advantage in that a sensitivity analysis was performed to confirm the robustness of the NMA results. We provided the NMA results with MD (not standardized MD) for applicability and interpretability in clinical practice.

However, this study has several limitations. First, the number of included studies and types of acupuncture modalities were relatively small. Heterogeneity exists between acupuncture regimens, even though we adopted a random-effects model. Therefore, further acupuncture RCTs on PD are needed to ensure the robustness of our results. In further NMA studies with more clinical RCTs, we can focus on more specific clinical questions, such as responders to acupuncture treatment in terms of severity, age, sex, disease duration, and accompanying symptoms [67]. In terms of dosage, we could not conduct a subgroup analysis of treatment duration, frequency, or needle retention time due to the lack of relevant studies. Since it is an important factor for the therapeutic effect of acupuncture [68,69], we need further subgroup analysis or meta-regression studies for detailed treatment regimens and dosages in acupuncture treatment. Second, in the sensitivity analysis, although this is largely consistent with the results of the primary analysis, the order of the effect sizes of ELEC and MANU was reversed in some cases. This suggests that it is difficult to differentiate between ELECs and MANUs. Further research is needed on this issue from an academic perspective. However, from a clinical perspective, it is recommended to combine electroacupuncture and MA simultaneously based on CM, as a commercial electroacupuncture device usually covers less than 12 acupuncture points. Third, we excluded combined acupuncture strategies, such as BVA combined with electroacupuncture, to explore the effect of a single acupuncture modality. However, in real-world clinical practice, each acupuncture modality is combined with other types of acupuncture. Therefore, we could not assess the synergetic effects of acupuncture modalities. Moreover, we might have underestimated the effects of acupuncture. Because the number of relevant RCTs was insufficient, further NMA studies are also needed on combined acupuncture modalities in the future. Next, the methodological quality of the included RCTs was relatively poor. Therefore, caution should be exercised when interpreting these results. Caution is also required when interpreting our results, as the reference group (CONV) of NMA had considerable heterogeneity. Finally, we included only CM in the reference (control) group. However, there are various standard treatments, such as surgical intervention and rehabilitation. As we used pharmacologic treatment as a control group, it might provide different results when using non-pharmacological intervention as a control group in the further NMA study.

## 5. Conclusions

We conducted a PMA and NMA to evaluate the effects of various acupuncture modalities on patients with idiopathic PD. The probability of comparative effectiveness in motor symptoms of patients with idiopathic PD was assumed to be in the order of BEEV, MANU, ELEC, SHAM, and CONV. However, more rigorous RCTs are needed for further NMA, including non-motor symptoms of PD. Along with conventional levodopa therapy, BVA, electroacupuncture, and MA could be more effective in clinical practice than single-drug therapy.

## Figures and Tables

**Table 1:** Characteristics of the included studies.

First Author, Year (Location)	Sample Size (A:B) (Initial →Final)	Age (Year), Mean ± SD	Sex (M:F)	Disease Severity : H-Y Stage	Disease Duration (Year)	(A) Treatment Intervention (Conventional Drug Therapy Dosage, mg/d)	(B) Control Intervention (Conventional Drug Therapy Dosage, mg/day)	Treatment/Follow-Up Period (Week)
Electroacupuncture + Conventional drug therapy								
Chen 2012 [14] (China)	30:30 →30:30	(A) 65.60 ± 3.79 (B) 61.93 ± 3.67	(A) 19:11 (B) 17:13	(A) 2.18 ± 0.26 (B) 2.04 ± 0.30	(A) 5.40 ± 1.75 (B) 6.40 ± 2.15	ELEC (432 ± 139)	CONV (435 ± 154)	6/None
Gu 2013 [48] (China)	23:25 →23:25	(A) 66 ± 8 (B) 70 ± 8	(A) 10:13 (B) 15:10	NR	(A) 4.44 ± 3.32 (B) 4.56 ± 3.11	ELEC (250)	CONV (250)	12/None
Huang 2009 [46] (China)	15:15 →15:15	(A) 65.60 ± 3.78 (B) 60.80 ± 3.63	(A) 8:7 (B) 6:9	(A) 2.18 ± 0.26 (B) 2.04 ± 0.30	(A) 5.40 ± 1.75 (B) 6.4 ± 2.14	ELEC (375−750)	CONV (375−750)	5/None
Lei 2016 [36] (USA)	10:5 →10:5	(A) 69.8 ± 4.5 (B) 71.0 ± 11.7	(A) 6:4 (B) 2:3	(A) 3.0 ± 1.0 (B) 2.9 ± 0.7	(A) 6.2 ± 5.9 (B) 5.2 ± 4.7	ELEC (614 ± 381)	SHAM (324 ± 295)	3/None
Liu 2016 [49] (China)	39:35 →39:35	(A) 65.65 ± 4.15 (B) 65.59 ± 4.18	(A) 21:18 (B) 19:16	NR	(A) 4.41 ± 2.01 (B) 4.33 ± 2.04	ELEC (NR)	CONV (NR)	12/None
Wang 2015 [40] (China)	30:20 →28:20	(A) 62.1 ± 8.7 (B) 59.1 ± 12.4	(A) 13:15 (B) 9:11	(A) 2.0 ± 0.7 (B) 2.0 ± 0.8	(A) 2.9 ± 2.9 (B) 2.7 ± 2.3	ELEC (104.1 ± 253.2)	CONV (160.6 ± 260.0)	Two months/ None
Xu 2020 [42] (China)	38:38 →33:37	(A) 61.73 ± 10.28 (B) 61.95 ± 9.77	(A) 15:18 (B) 21:16	Stage 1 (A) 9 (B) 8 Stage 1.5 (A) 4 (B) 12 Stage 2 (A) 5 (B) 6 Stage 2.5 (A) 9 (B) 5 Stage 3 (A) 4 (B) 4 Stage 4 (A) 2 (B) 2	(A) 3.52 ± 2.78 (B) 3.26 ± 2.32	ELEC (187.5−375)	CONV (187.5−375)	8/4
MA + Conventional drug therapy								
Jung 2006 [15] (Korea)	NR →16:21	(A) 59.69 ± 9.6 (B) 61.00 ± 9.7	(A) 11:5 (B) 10:11	NR	(A) 5.66 ± 4.23 (B) 6.07 ± 4.82	MANU (NR)	SHAM (NR)	4/None
Kluger 2016 [43] (USA)	47:47 →47:47	(A) 64.4 ± 10.3 (B) 63.0 ± 13.0	(A) 30:17 (B) 29:18	Stage 1 (A) 4 (B) 2 Stage 1.5 (A) 6 (B) 3 Stage 2 (A) 11 (B) 17 Stage 2.5 (A) 18 (B) 12 Stage 3 (A) 6 (B) 10 Stage 4 (A) 0 (B) 4	NR	MANU (558.9 ± 379.3)	SHAM (628.6 ± 482.9)	6/None
Kong 2017 [37] (Singapore)	20:20 →19:17	(A) 66.4 ± 6.5 (B) 62.9 ± 9.7	(A) 6:14 (B) 7:13	NR	(A) 87.2 ± 53.2 (B) 50.1 ± 26.4	MANU (637.8 ± 394.3)	SHAM (592.6 ± 303.1)	5/4
Li 2018 [38] (China)	14:13:14 →14:12:11	(A) 62.17 ± 7.66 (B) 65.79 ± 6.07 (C) 62.85 ± 5.00	(A) 9:3 (B) 8:6 (C) 7:6	NR	NR	(A) MANU (367.86 ± 146.24)	(B) SHAM(338.46 ± 112.09) (C) CONV (345.83 ± 173.81)	12/None
Lu 2020 [50] (China)	20:20 →20:20	(A) 66.50 ± 8.81 (B) 65.90 ± 8.92	(A) 10:10 (B) 12:8	NR	(A) 15.10 ± 1.72 (B) 15.25 ± 2.04	MANU (250)	CONV (250)	4/None
Mizushima 2011 [44] (Japan)	NR →103:95	(A) 63.9 ± 8.2 (B) 64.7 ± 9.8	(A) 45:58 (B) 50:45	NR	(A) 1.6 ± 0.6 (B) 1.8 ± 1.2	MANU (186.0 ± 134.0)	CONV (251.0 ± 172.8)	Five years/5 years
Park 2007 [45] (Korea)	NR →21:13	(A) 60.00 ± 9.0 (B) 61.26 ± 9.81	(A) 12:9 (B) 2:11	(A) 1.7619 ± 0.95 (B) 1.8846 ± 0.68	(A) 5.63 ± 5.29 (B) 5.84 ± 3.3	MANU (NR)	SHAM (NR)	4/None
Ren 2011 [47] (China)	90:90 →90:90	(A) 59.1 ± 12.1 (B) 58.2 ± 11.9	(A) 52:38 (B) 49:41	NR	(A) 1.8 ± 0.3 (B) 1.9 ± 0.4	MANU (750)	CONV (750)	30 days/None
BVA + Conventional drug therapy								
Cho 2012 [41] (Korea)	18:17:14→13:13:9	(A) 57.0 (B) 55.0 (C) 57.0	(A) 5:8 (B) 5:8 (C) 3:6	NR	(A) 5.0 (B) 6.0 (C) 5.0	(A) BEEV (NR) (B) MANU (NR)	(C) CONV (NR)	8/None
Hartmann 2016 [39] (France)	20:20 →15:20	(A) 60.3 ± 15 (B) 63.3 ± 8	(A) 8:12 (B) 12:8	Stage 2 (A) 6 (B) 7 Stage 2.5(A) 14 (B) 11 Stage 3 (A) 0 (B) 2	(A) 6.2 ± 5 (B) 6.3 ± 5.1	BEEV (391→64 ± 127) Baseline median→Result mean, SD	SHAM (512→98 ± 156) Baseline median→Result mean, SD	11 months

(A) Treatment intervention; (B) Control intervention (in 2 arm design); (C) Control intervention in 3 arm design; SD, standard deviation; H-Y stage, Hoehn and Yahr stage; NR, not reported. Intervention: ELEC, electroacupuncture + conventional drug therapy; SHAM, sham acupuncture + conventional drug therapy; MANU, manual acupuncture + conventional drug therapy; CONV, single conventional drug therapy; BEEV, bee-venom acupuncture + conventional drug therapy. Outcomes) UPDRS: Unified PD rating scale; AE: Rate of the number of participants with adverse events between groups; DR, dropout rate. Li 2018: MANU vs. SHAM vs. CONV; Cho 2012: BEEV vs. MANU vs. CONV.

**Table 2:** Detailed description of the acupuncture treatment.

First Author, Year (Location)	Acupuncture Point	Depth of Insertion	Needle Stimulation, Electrical Stimulation	Needle Retention Time	Treatment Frequency, Total Number of Treatment Session	Duration of Treatment Sessions
Electroacupuncture + Conventional drug therapy						
Chen 2012 [14] (China)	GV20, EX-HN1, EX-HN3	NR	2 Hz frequency	1 h	3 times a week, 18 total sessions	6 weeks
Gu 2013 [48] (China)	Bilateral anterior parietal-temporal oblique lines (motor areas) GB20, LI11, LI4, LR3, KI3, GB34	NR	2 Hz frequency, The strength the patient can tolerate	20 min	3 times a week, 36 total sessions	12 weeks
Huang 2009 [46] (China)	MS6, MS4, MS8, MS9, MS14	NR	Continuous waves, 100 Hz frequency, 2−4 mA	30 min	6 times a week, 30 total sessions	5 weeks
Lei 2016 [36] (USA)	Foot motor sensory area, balance area, GV20, GV14, LI4, ST36, GB34, BL40, SP6, KI3, LR3	NR	Amplitude (intensity) 3.5 and 4.5, Frequency 100 Hz or 4 Hz	30 min	Once a week, 3 total sessions	3 weeks
Liu 2016 [49] (China)	Anterior parietal and temporal oblique lines (motor areas) on both sides, LR3, KI3, LI11, GB20, GB34, LI4	NR	2 Hz frequency, The strength the patient can tolerate	20 min	Three times a week, 36 total sessions	12 weeks
Wang 2015 [40] (China)	Bilateral GB20, LI4, Central Du14, Du16	2−2.5 cm	Pulses of 9 V, 1 A, 9 W, 100 Hz	30 min	Once every three days, 20 total sessions	2 months
Xu 2020 [42] (China)	GV17, GB19, Sishenzhen, and temporal three-needle	0.8−1.5 cm	Twisting, lifting and thrusting, continuous waves at alternating low 100 Hz frequency	30 min	Four days per week, 32 total sessions	8 weeks
MA + Conventional drug therapy						
Cho 2012 [41] (Korea)	Bilateral GB20, LI11, GB34, ST36, LR3	1.0−1.5 cm	Twisting at 2 Hz for 10 s	20 min	Twice a week, 16 total sessions	8 weeks
Jung 2006 [15] (Korea)	Bilateral LR3, GB34	NR	None	15 min	Twice a week, 8 total sessions	4weeks
Kluger 2016 [43] (USA)	GV20, GV24, CV6, Bilateral LI10, HT7, ST36, SP6	0.5−1 cm	Twisting three times in a clockwise direction	30 min	Twice a week (at least one day apart), 12 total sessions	6 weeks
Kong 2017 [37] (Singapore)	Bilateral PC6, LI4, ST36, SP6, KI3, CV6	0.5−1 inch	None	20 min	Twice a week (at least three days apart), 10 total sessions	5 weeks
Li 2018 [38] (China)	DU20, Bilateral GB20, Chorea-Tremor Controlled Zone	2−3 cm	Twist every 10 min	30 min	Twice a week, 24 total sessions	12 weeks
Lu 2020 [50] (China)	LR3, LR2, LR8, KI3, KI7, KI10, SP6, ST36, LI11, PC6, LI4	20−30 mm	After 15 min, the needle is lifted, inserted, and twisted once	30 min	Once a day, 28 total sessions	28 days
Mizushima 2011 [44] (Japan)	Individualized point according to meridian diagnosis	Individualized depth	Individualized way according to diagnosis	NR	Two to four times a month, NR	5 years
Park 2007 [45] (Korea)	One side LR3, GB34, ST36	NR	None	15 min	Twice a week, 8 total sessions	4 weeks
Ren 2011 [47] (China)	Bilateral BL18, BL23, GB20, LI11, LI4, GB34, KI3, LR3	NR	Flattening and relieving	30 min	Once a day, 30 total sessions	30 days
BVA + Conventional drug therapy						
Cho 2012 [41] (Korea)	Bilateral GB20, LI11, GB34, ST36, LR3	NR	0.1 mL BEEV diluted to 0.005% in distilled water	-	Twice a week, 16 total sessions	8 weeks
Hartmann 2016 [39] (France)	NR	s.c	BEEV (Alyostal® 100 μg in 1 mL of NaCl 0.9%)	-	Once a month, 11 total sessions	11 months

NR, Not reported; SC, subcutaneous; The control group, including the placebo acupuncture group, received the same frequency and total number of acupuncture treatments as the treatment group.

**Table 3:** League table on UPDRS-III.

**BEEV**	−3.31(−8.91;2.29)		−7.13(−16.98;2.72)	−5.44(−12.53;1.65)
−3.24(−7.72;1.23)	**MANU**		−1.41(−4.38;1.57)	**−4.07(−5.72;−2.41;)**
−3.71(−9.01;1.59)	−0.47(−3.57;2.64)	**ELEC**	-	**−3.66(−6.29;−1.03)**
−4.66(−9.63;0.31)	−1.42(−4.24;1.41)	−0.95(−5.10;3.20)	**SHAM**	−7.48(−17.54;2.58)
**−7.37(−11.97;−2.77)**	**−4.13(−5.78;−2.47)**	**−3.66(−6.29;−1.03)**	−2.71(−5.92;0.50)	**CONV**

BEEV, bee venom acupuncture + conventional drug therapy; CONV, single conventional drug therapy; ELEC, electroacupuncture + conventional drug therapy; MANU, manual acupuncture + conventional drug therapy; SHAM, sham acupuncture + conventional drug therapy; UPDRS, Unified PD rating scale.

## Data Availability

Data are available on request to the corresponding author. The study protocol is available at the open science platform (https://osf.io/q8n7z/).

## Supplementary Materials

The following are available online at https://www.mdpi.com/article/10.3390/healthcare9111502/s1. Supplemental Digital Content 1. Search strategies used on each database. Supplemental Digital Content 2. Articles excluded after the full text review with reasons. Supplemental Digital Content 3. Risk of bias graph. Supplemental Digital Content 4. Forest plot of PMA (UPDRS-III and UPDRS-II). Supplemental Digital Content 5. Net-split plot: UPDRS-III score. Supplemental Digital Content 6. Sensitivity analysis. Supplemental Digital Content 7. Network plot, forest plot, league table, net-split plot, and funnel plot on the secondary outcome (UPDRS-II).
