# Supplementary material for: Effect of Acupuncture on Movement Function in Patients with Parkinson’s Disease: Network Meta-Analysis of Randomized Controlled Trials
Source: Healthcare (Basel). 2021 Nov 5;9(11):1502. doi: 10.3390/healthcare9111502 (PMC8619200; doi:10.3390/healthcare9111502)
Supplement: Supplementary file 1 [file healthcare-09-01502-s001.zip › Supplement Digital Content 1_Search strategy.pdf]

Supplementary Digital Contents1. Search strategies used in each Database

1A. Search strategies used in MEDLINE via PubMed

	Searches	Results
#1	"parkinson disease"[Mesh]	68,299
#2	parkinson disease OR Idiopathic Parkinson's Disease OR Lewy Body Parkinson's Disease OR Parkinson's Disease, Idiopathic OR Parkinson's Disease, Lewy Body OR Parkinson Disease, Idiopathic OR Parkinson's Disease OR Idiopathic Parkinson Disease OR Lewy Body Parkinson Disease OR Primary Parkinsonism OR Parkinsonism, Primary OR Paralysis Agitans	119,065
#3	"acupuncture" [Mesh]	1,717
#4	Acupuncture OR Pharmacopuncture OR Acupuncture Treatment OR Acupuncture Treatments OR Treatment, Acupuncture OR Therapy, Acupuncture OR Pharmacoacupuncture Treatment OR Treatment, Pharmacoacupuncture OR Pharmacoacupuncture Therapy OR Therapy, Pharmacoacupuncture OR Acupotomy OR Acupotomies	34,516
#5	Electroacupuncture	6,014
#6	Bee Venoms OR Venoms, Bee OR Bee Venom OR Venom, Bee OR Apis Venoms OR Venoms, Apis OR Apitoxin OR Honeybee Venoms OR Venoms, Honeybee OR Honeybee Venom OR Venom, Honeybee	6,832
#7	Fire needle therapy OR Fire acupuncture	231
#8	(#1 OR #2) AND (#3 OR #4 OR #5 OR #6 OR #7)	345

1B Search strategies used in EMBASE

	Searches	Results
#1	'parkinson disease'/exp	164,234
#2	'Parkinson disease' OR 'Parkinson Disease, Lewy Body' OR 'Parkinson Disease, Idiopathic' OR 'Idiopathic Parkinson Disease' OR 'Lewy Body Parkinson Disease' OR 'Primary Parkinsonism' OR 'Parkinsonism, Primary'	167,444

	OR 'Paralysis Agitans'	
#3	'acupuncture'/exp	48,475
#4	'Acupuncture' OR 'Pharmacopuncture' OR 'Acupuncture Treatment' OR 'Acupuncture Treatments' OR 'Treatment, Acupuncture' OR 'Therapy, Acupuncture' OR 'Pharmacoacupuncture Treatment' OR 'Treatment, Pharmacoacupuncture' OR 'Pharmacoacupuncture Therapy' OR 'Therapy, Pharmacoacupuncture' OR 'Acupotomy' OR 'Acupotomies'	53,475
#5	Electroacupuncture	8,283
#6	'Bee Venoms' OR 'Venoms, Bee' OR 'Bee Venom' OR 'Venom, Bee' OR 'Apis Venoms' OR 'Venoms, Apis' OR 'Apitoxin' OR 'Honeybee Venoms' OR 'Venoms, Honeybee' OR 'Honeybee Venom' OR 'Venom, Honeybee'	4,673
#7	'Fire needle therapy' OR 'Fire acupuncture'	47
#8	(#1 OR #2) AND (#3 OR #4 OR #5 OR #6 OR #7)	538

1C Search strategies used in Cochrane

	Searches	Results
#1	MeSH descriptor: [parkinson disease] explode all trees	4,233
#2	parkinson disease OR Idiopathic Parkinson's Disease OR Lewy Body Parkinson's Disease OR Parkinson's Disease, Idiopathic OR Parkinson's Disease, Lewy Body OR Parkinson Disease, Idiopathic OR Parkinson's Disease OR Idiopathic Parkinson Disease OR Lewy Body Parkinson Disease OR Primary Parkinsonism OR Parkinsonism, Primary OR Paralysis Agitans	10,683
#3	MeSH descriptor: [acupuncture] explode all trees	151
#4	Acupuncture OR Pharmacopuncture OR Acupuncture Treatment OR Acupuncture Treatments OR Treatment, Acupuncture OR Therapy, Acupuncture OR Pharmacoacupuncture Treatment OR Treatment, Pharmacoacupuncture OR Pharmacoacupuncture Therapy OR Therapy, Pharmacoacupuncture OR Acupotomy OR Acupotomies	16,921
#5	Electroacupuncture	2,670

#6	Bee Venoms OR Venoms, Bee OR Bee Venom OR Venom, Bee OR Apis Venoms OR Venoms, Apis OR Apitoxin OR Honeybee Venoms OR Venoms, Honeybee OR Honeybee Venom OR Venom, Honeybee	155
#7	Fire needle therapy OR Fire acupuncture	190
#8	(#1 OR #2) AND (#3 OR #4 OR #5 OR #6 OR #7) in Trials	115

1D Search strategies used in CNKI

	Searches	Results
#1	Parkinson AND Acupuncture	663
#2	Parkinson AND Pharmacopuncture	9
#3	Parkinson AND Electroacupuncture	245
#4	Parkinson AND Bee venom	57
#5	Parkinson AND Fire acupuncture	11
#6	#1 OR #2 OR #3 OR #4 OR #5	755

1E Search strategies used in OASIS

	Searches	Results
#1	Parkinson AND Acupuncture(Title, Abstract, Keywords)	120
#2	Parkinson AND Pharmacopuncture(Title, Abstract, Keywords)	12
#3	Parkinson AND Electroacupuncture(Title, Abstract, Keywords)	6
#4	Parkinson AND Bee venom(Title, Abstract, Keywords)	9
#5	Parkinson AND Fire acupuncture(Title, Abstract, Keywords)	0
#6	#1 OR #2 OR #3 OR #4 OR #5	78

1F Search strategies used in RISS

	Searches	Results
#1	Parkinson AND Acupuncture	98
#2	Parkinson AND Pharmacopuncture	20
#3	Parkinson AND Electroacupuncture	7
#4	Parkinson AND Bee venom	22
#5	Parkinson AND Fire acupuncture	0
#6	#1 OR #2 OR #3 OR #4 OR #5	105

1G Search strategies used in KCI

	Searches	Results
#1	Parkinson AND Acupuncture	85
#2	Parkinson AND Pharmacopuncture	17
#3	Parkinson AND Electroacupuncture	5
#4	Parkinson AND Bee venom	17
#5	Parkinson AND Fire acupuncture	1
#6	#1 OR #2 OR #3 OR #4 OR #5	107

1H Search strategies used in NDSL

	Searches	Results
#1	Parkinson AND Acupuncture	85
#2	Parkinson AND Pharmacopuncture	8
#3	Parkinson AND Electroacupuncture	5
#4	Parkinson AND Bee venom	19
#5	Parkinson AND Fire acupuncture	1
#6	#1 OR #2 OR #3 OR #4 OR #5	88
