# Supplementary material for: Effect of Acupuncture on Movement Function in Patients with Parkinson’s Disease: Network Meta-Analysis of Randomized Controlled Trials
Source: Healthcare (Basel). 2021 Nov 5;9(11):1502. doi: 10.3390/healthcare9111502 (PMC8619200; doi:10.3390/healthcare9111502)
Supplement: Supplementary file 1 [file healthcare-09-01502-s001.zip › Supplement Digital Content 2_Excluded study.pdf]

Supplemental Digital Contents 2. Articles excluded after full-text reviewing with reasons

Study	Reason
Aroxa 2017[1]	UPDRS is not used
Chae 2009[2]	Multiple intervention
Chen 2015[3]	Not RCT
Cho 2018[4]	Multiple intervention
Cristian 2005[5]	UPDRS is not used
Doo 2015[6]	Not RCT
Eng 2006[7]	Not RCT
Fukuda 2016[8]	Not RCT
Huang 2009[9]	UPDRS is not used
Huang 2010[10]	UPDRS is not used
Jang 2020[11]	MDS UPDRS
Ren 2008[12]	Not RCT
Shulman 2002[13]	Inappropriate experimental group
Toosizadeh 2015[14]	Duplication
Wang 2002[15]	UPDRS is not used
Yeo 2014[16]	Not RCT
Yeo 2012[17]	Not RCT
Yeo 2018[18]	Not RCT

Yu 2019[19]	Not RCT
Zhuang 2000[20]	Not RCT
Jiang 2006[21]	No UPDRSIII results
Zhuo 2016[22]	UPDRSII,III scores are marked as a sum
Luo 2019[23]	No reports on basic treatment for Parkinson's disease. (Phenolphthalein was used)
Lin 2018[24]	The basic treatment for Parkinson's disease is not specified exactly.
Zhang 2020[25]	UPDRS scores are marked as a sum
Zhang 2013[26]	Multiple intervention. Gastrodin was used in both groups.
Feng 2011[27]	Duplication
LIU 2015[28]	No UPDRSIII results

Appendix 16 Reference

1. Aroxa FH de A, Gondim ITG de O, Santos ELW, Coriolano M das GW de S, Asano AGC, Asano NMJ. Acupuncture as Adjuvant Therapy for Sleep Disorders in Parkinson's Disease. J Acupunct Meridian Stud. 2017;10(1):33-38.

2. Chae Y, Lee H, Kim H, et al. Parsing brain activity associated with acupuncture treatment in Parkinson's diseases. Mov Disord. 2009;24(12):1794-1802.

3. Chen F-P, Chang C-M, Shiu J-H, et al. A clinical study of integrating acupuncture and Western medicine in treating patients with Parkinson's disease. Am J Chin Med. 2015;43(3):407-423.

4. Cho S-Y, Lee Y-E, Doo K-H, et al. Efficacy of Combined Treatment with Acupuncture and Bee Venom Acupuncture as an Adjunctive Treatment for Parkinson's Disease. J Altern Complement Med. 2018;24(1):25-32.

5. Cristian A, Katz M, Cutrone E, Walker RH. Evaluation of acupuncture in the treatment of Parkinson's disease: a double-blind pilot study. Mov Disord. 2005;20(9):1185-1188.

6. Doo K-H, Lee J-H, Cho S-Y, et al. A Prospective Open-Label Study of Combined Treatment for Idiopathic Parkinson's Disease Using Acupuncture and Bee Venom Acupuncture as an Adjunctive Treatment. J Altern Complement Med. 2015;21(10):598-603.

7. Eng ML, Lyons KE, Greene MS, Pahwa R. Open-label trial regarding the use of acupuncture and yin tui na in Parkinson's disease outpatients: a pilot study on efficacy, tolerability, and quality of life. J Altern Complement Med. 2006;12(4):395-399.

8. Fukuda S, Kuriyama N, Tsuru H, Egawa M. Immediate effects of acupuncture on tongue pressure including swallowing reflex latency in Parkinson's disease. Acupunct Med. 2016;34(1):59-61.

9. Huang Y, Jiang X, Zhuo Y, Tang A, Wik G. Complementary acupuncture treatment increases cerebral metabolism in patients with Parkinson's disease. Int J Neurosci. 2009;119(8):1190-1197.

10. Huang Y, Jiang X, Zhuo Y, Wik G. Complementary acupuncture in Parkinson's disease: a spect study. Int J Neurosci. 2010;120(2):150-154.

11. Jang J-H, Park S, An J, et al. Gait Disturbance Improvement and Cerebral Cortex Rearrangement by Acupuncture in Parkinson's Disease: A Pilot Assessor-Blinded, Randomized, Controlled, Parallel-Group Trial. Neurorehabil Neural Repair. 2020;34(12):1111-1123.

12. Ren X. Fifty cases of Parkinson's disease treated by acupuncture combined with madopar. J Tradit Chin Med. 2008;28(4):255-257.

13. Shulman LM, Wen X, Weiner WJ, et al. Acupuncture therapy for the symptoms of Parkinson's disease. Mov Disord. 2002;17(4):799-802.

14. Toosizadeh N, Lei H, Schwenk M, et al. Does integrative medicine enhance balance in aging adults? Proof of concept for the benefit of electroacupuncture therapy in Parkinson's disease. Gerontology. 2015;61(1):3-14.

15. Wang L, He C, Liu Y, Zhu L. Effect of acupuncture on the auditory evoked brain stem potential in Parkinson's disease. J Tradit Chin Med. 2002;22(1):15-17.

16. Yeo S, Choe I-H, van den Noort M, et al. Acupuncture on GB34 activates the

precentral gyrus and prefrontal cortex in Parkinson's disease. BMC Complement Altern Med. 2014;14:336.

17. Yeo S, Lim S, Choe I-H, et al. Acupuncture stimulation on GB34 activates neural responses associated with Parkinson's disease. CNS Neurosci Ther. 2012;18(9):781-790.

18. Yeo S, van den Noort M, Bosch P, Lim S. A study of the effects of 8-week acupuncture treatment on patients with Parkinson's disease. Medicine (Baltimore). 2018;97(50):e13434.

19. Yu S-W, Lin S-H, Tsai C-C, et al. Acupuncture Effect and Mechanism for Treating Pain in Patients With Parkinson's Disease. Front Neurol. 2019;10:1114.

20. Zhuang X, Wang L. Acupuncture treatment of Parkinson's disease--a report of 29 cases. J Tradit Chin Med. 2000;20(4):265-267.

21. Jiang X, Huang Y, Zhuo Y, Gao Y. [Therapeutic effect of scalp electroacupuncture on Parkinson disease]. Nan Fang Yi Ke Da Xue Xue Bao. 2006;26(1):114-116.

22. 周琛, 庞伟, 吴岚. 针灸联合美多芭治疗帕金森病的临床效果和安全性分析. 中国医药科学. 2016;6(22):11-13+18.

23. 罗思宁, 张艳. 电针足三里联合常规西药治疗帕金森伴便秘的临床效果. 临床医学研究与实践. 2019;4(27):136-138.

24. 林铎, 赖新生, 庄子齐, 杨楠. 针刺四关穴治疗帕金森病剂末现象的临床观察. 浙江中医药大学学报. 2018;42(02):157-159.

25. 张燕平, 刘钢敏, 李燕梅. 针刺配合盐酸普拉克索对帕金森病伴抑郁疗效观察. 北京中医药. 2020;39(06):594-597.

26. 张小莉, 冯为菊, 陈义良, 罗阳, 王红磊. 针药并用治疗帕金森病合并轻度认知损害疗效观察. 上海针灸杂志. 2013;32(04):263-265.

27. 冯为菊, 陈义良, 张小莉. 针刺联合小剂量托特罗定治疗帕金森病合并膀胱过度活动症. 中国实用神经疾病杂志. 2011;14(16):7-10.

28. 刘承浩, 王睿, 金亚蓓, 孙占玲, 周翔, 何嘉莹. 葛根素穴位注射治疗早中期帕金森病: 多中心随机对照试验. 针刺研究. 2015;40(01):56-60.
