# Supplementary material for: Effect of Acupuncture on Movement Function in Patients with Parkinson’s Disease: Network Meta-Analysis of Randomized Controlled Trials
Source: Healthcare (Basel). 2021 Nov 5;9(11):1502. doi: 10.3390/healthcare9111502 (PMC8619200; doi:10.3390/healthcare9111502)
Supplement: Supplementary file 1 [file healthcare-09-01502-s001.zip › Supplement Digital Content 3_ROB.pdf]

Supplemental Digital Contents 3. Risk of bias graph

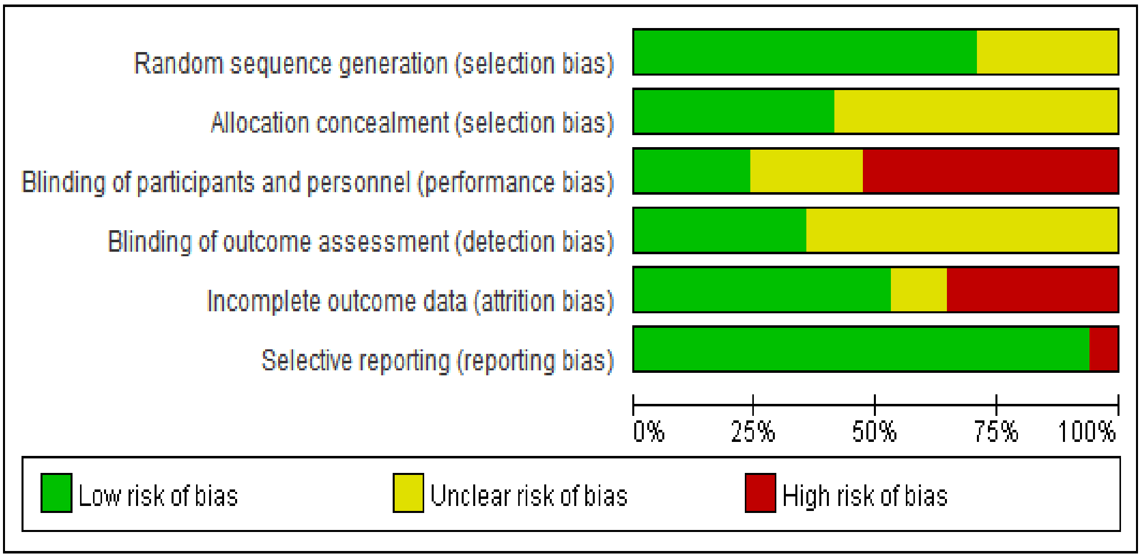

	Random sequence generation (selection bias)	Allocation concealment (selection bias)	Blinding of participants and personnel (performance bias)	Blinding of outcome assessment (detection bias)	Incomplete outcome data (attrition bias)	Selective reporting (reporting bias)
Chen 2012	+	?	−	?	+	+
Cho 2012	+	+	−	+	−	+
Gu 2013	?	?	−	?	+	+
Hartmann 2016	+	+	+	?	−	+
Huang 2009	?	?	−	?	+	+
Jung 2006	+	?	+	?	?	+
Kluger 2016	+	+	−	?	+	+
Kong 2017	+	+	+	+	−	+
Lei 2016	?	?	?	+	−	−
Li 2018	+	+	−	+	−	+
Liu 2016	?	?	−	?	+	+
Lu 2020	+	?	−	?	+	+
Mizushima 2011	+	?	?	?	?	+
Park 2007	+	?	+	?	−	+
Ren 2011	+	+	?	?	+	+
Wang 2015	?	?	?	+	+	+
Xu 2020	+	+	−	+	+	+
