# Supplementary material for: Effect of Acupuncture on Movement Function in Patients with Parkinson’s Disease: Network Meta-Analysis of Randomized Controlled Trials
Source: Healthcare (Basel). 2021 Nov 5;9(11):1502. doi: 10.3390/healthcare9111502 (PMC8619200; doi:10.3390/healthcare9111502)

Supplemental Digital Contents 4. Forest plot of pairwise meta-analysis (UPDRS III and UPDRS II)

4.1 Acupuncture + Conventional drug therapy vs Conventional drug therapy, UPDRSIII score

Study or Subgroup	Acupuncture+CONV Mean	Acupuncture+CONV SD	Acupuncture+CONV Total	CONV Mean	CONV SD	CONV Total	Weight	Mean Difference IV, Random, 95% CI
**1.1.1 Electroacupuncture**								
Chen 2012	29.98	8.26	30	33.58	8.59	30	32.2%	-3.60 [-7.86, 0.66]
Gu 2013	29.04	10.62	23	33.12	17.42	25	8.9%	-4.08 [-12.17, 4.01]
Huang 2009	32.8	12.6	15	38.6	12.54	15	7.2%	-5.80 [-14.80, 3.20]
Liu 2016	28.95	10.59	39	32.24	15.68	35	15.4%	-3.29 [-9.46, 2.88]
Wang 2015	20.6	2.7	28	22.5	12.1	20	20.1%	-1.90 [-7.30, 3.50]
Xu 2020	23.88	13.73	33	28.84	11.68	37	16.2%	-4.96 [-10.97, 1.05]
**Subtotal (95% CI)**			**168**			**162**	**100.0%**	**-3.63 [-6.05, -1.21]**
Heterogeneity: Tau² = 0.00; Chi² = 0.83, df = 5 (P = 0.98); I² = 0%								
Test for overall effect: Z = 2.94 (P = 0.003)								
**1.1.2 Manual Acupuncture**								
Cho 2012	13.31	7.79	13	15.44	9.28	9	7.9%	-2.13 [-9.53, 5.27]
Li 2018	21.64	13.68	14	26.73	14.03	11	4.0%	-5.09 [-16.05, 5.87]
Lu 2020	17.55	4.17	20	18.05	3.9	20	26.0%	-0.50 [-3.00, 2.00]
Mizushima 2011	11.9	6.8	103	18.2	9.8	95	26.8%	-6.30 [-8.67, -3.93]
Ren 2011	11.73	2.94	90	16.58	3.08	90	35.2%	-4.85 [-5.73, -3.97]
**Subtotal (95% CI)**			**240**			**225**	**100.0%**	**-3.90 [-6.24, -1.57]**
Heterogeneity: Tau² = 3.83; Chi² = 13.03, df = 4 (P = 0.01); I² = 69%								
Test for overall effect: Z = 3.28 (P = 0.001)								
**1.1.3 Bee Venom Acupuncture**								
Cho 2012	10	5.23	13	15.44	9.28	9	100.0%	-5.44 [-12.14, 1.26]
**Subtotal (95% CI)**			**13**			**9**	**100.0%**	**-5.44 [-12.14, 1.26]**
Heterogeneity: Not applicable								
Test for overall effect: Z = 1.59 (P = 0.11)								
Test for subgroup differences: Chi² = 0.25, df = 2 (P = 0.88), I² = 0%								

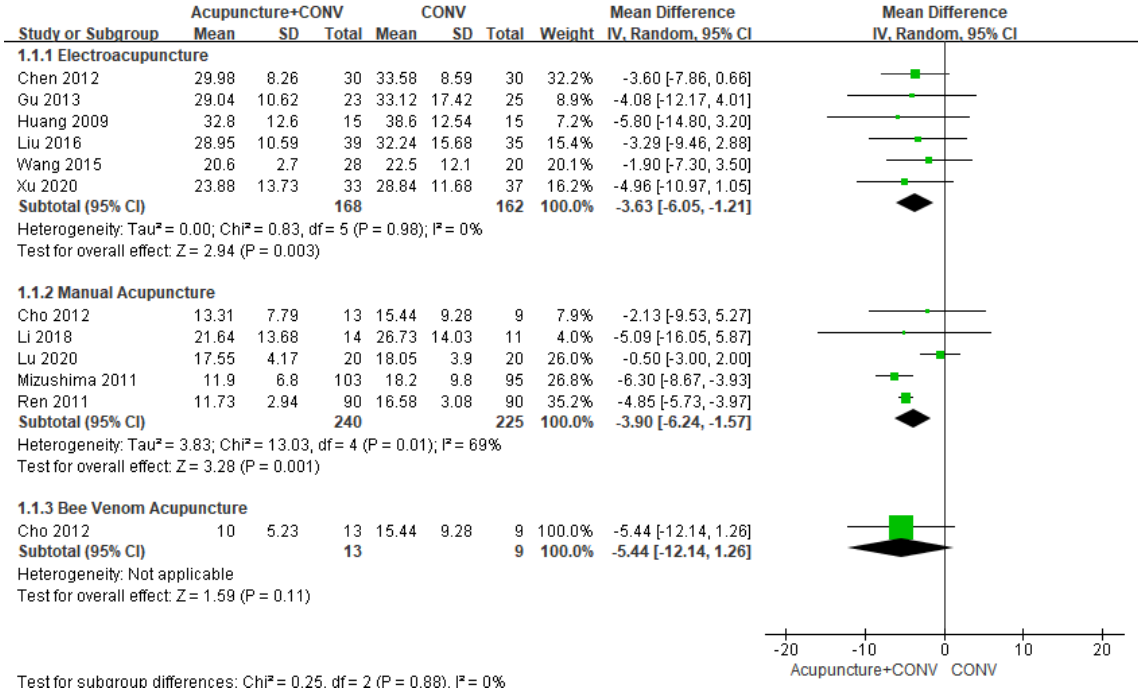

4.2 Acupuncture + Conventional drug therapy vs Sham Acupuncture + Conventional drug therapy, UPDRSIII score

Study or Subgroup	Acupuncture+CONV Mean	Acupuncture+CONV SD	Acupuncture+CONV Total	SHAM+CONV Mean	SHAM+CONV SD	SHAM+CONV Total	Weight	Mean Difference IV, Random, 95% CI
**2.1.1 Electroacupuncture**								
Lei 2016	19.1	10.9	10	37.2	11.6	5	7.7%	-18.10 [-30.31, -5.89]
**Subtotal (95% CI)**			**10**			**5**	**7.7%**	**-18.10 [-30.31, -5.89]**
Heterogeneity: Not applicable								
Test for overall effect: Z = 2.91 (P = 0.004)								
**2.1.2 Manual Acupuncture**								
Jung 2006	12.44	7.99	16	10.86	9.07	21	19.3%	1.58 [-3.93, 7.09]
Kluger 2016	21.3	8.8	47	25.9	11.7	47	23.1%	-4.60 [-8.79, -0.41]
Kong 2017	22.5	12.1	19	23.7	10.9	17	14.5%	-1.20 [-8.71, 6.31]
Li 2018	21.64	13.68	14	19.25	9.21	12	12.0%	2.39 [-6.47, 11.25]
Park 2007	17.86	15.13	21	18.69	10.08	13	12.6%	-0.83 [-9.31, 7.65]
**Subtotal (95% CI)**			**117**			**110**	**81.5%**	**-1.55 [-4.32, 1.21]**
Heterogeneity: Tau² = 0.20; Chi² = 4.07, df = 4 (P = 0.40); I² = 2%								
Test for overall effect: Z = 1.10 (P = 0.27)								
**2.1.3 Bee Venom Acupuncture**								
Hartmann 2016	25.27	12.8	15	32.4	16.09	20	10.8%	-7.13 [-16.71, 2.45]
**Subtotal (95% CI)**			**15**			**20**	**10.8%**	**-7.13 [-16.71, 2.45]**
Heterogeneity: Not applicable								
Test for overall effect: Z = 1.46 (P = 0.14)								
**Total (95% CI)**			**142**			**135**	**100.0%**	**-2.91 [-6.80, 0.98]**
Heterogeneity: Tau² = 12.48; Chi² = 11.65, df = 6 (P = 0.07); I² = 49%								
Test for overall effect: Z = 1.47 (P = 0.14)								
Test for subgroup differences: Chi² = 7.59, df = 2 (P = 0.02), I² = 73.7%								

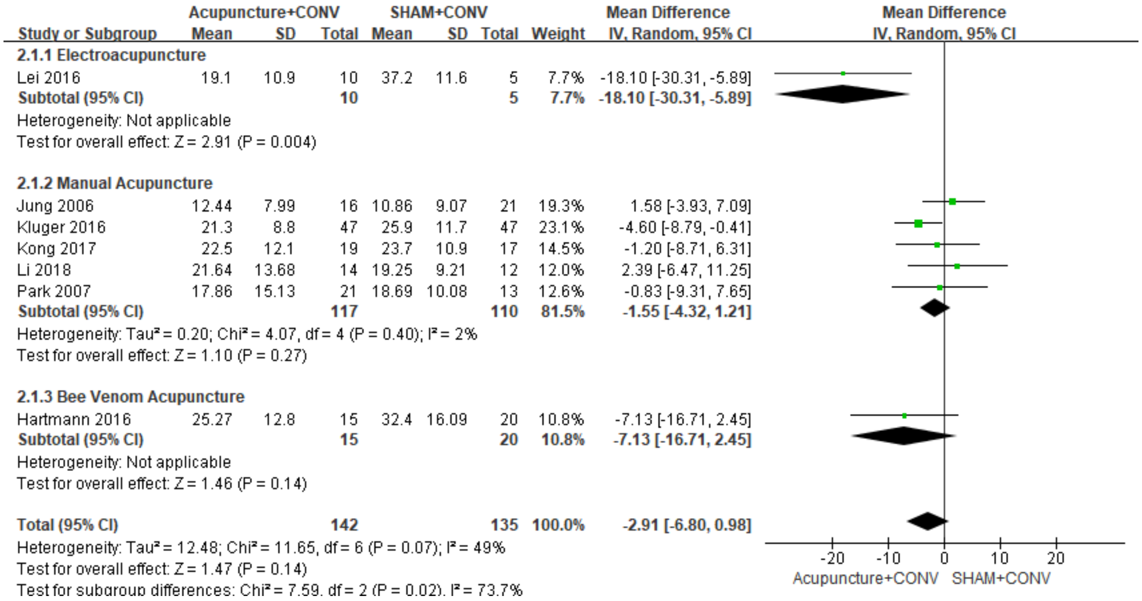

4.3 Acupuncture + Conventional drug therapy vs Conventional drug therapy, UPDRSII score

Study or Subgroup	Acupuncture+CONV Mean	SD	Total	CONV Mean	SD	Total	Weight	Mean Difference IV, Random, 95% CI
**1.2.1 Electoacupuncture**								
Gu 2013	8.61	3.78	23	13.96	6.37	25	33.4%	-5.35 [-8.29, -2.41]
Liu 2016	8.52	3.84	39	12.57	6.28	35	49.8%	-4.05 [-6.45, -1.65]
Xu 2020	23.06	7.25	33	27.19	10.3	37	16.8%	-4.13 [-8.27, 0.01]
**Subtotal (95% CI)**			**95**			**97**	**100.0%**	**-4.50 [-6.19, -2.80]**
Heterogeneity: Tau² = 0.00; Chi² = 0.49, df = 2 (P = 0.78); I² = 0%								
Test for overall effect: Z = 5.20 (P < 0.00001)								
**1.2.2 Manual acupuncture**								
Cho 2012	10.77	5.05	13	11.22	6.4	9	2.5%	-0.45 [-5.45, 4.55]
Li 2018	9	4.4	14	12.27	7.9	11	2.3%	-3.27 [-8.48, 1.94]
Lu 2020	25.9	3.45	20	28.5	4.38	20	10.5%	-2.60 [-5.04, -0.16]
Mizushima 2011	7.6	5	103	12.2	7.2	95	20.3%	-4.60 [-6.34, -2.86]
Ren 2011	8.72	2.82	90	13.03	3.56	90	64.3%	-4.31 [-5.25, -3.37]
**Subtotal (95% CI)**			**240**			**225**	**100.0%**	**-4.07 [-4.87, -3.27]**
Heterogeneity: Tau² = 0.03; Chi² = 4.10, df = 4 (P = 0.39); I² = 2%								
Test for overall effect: Z = 9.97 (P < 0.00001)								
**1.2.3 Bee Venom Acupuncture**								
Cho 2012	7.69	4.37	13	11.22	6.4	9	100.0%	-3.53 [-8.34, 1.28]
**Subtotal (95% CI)**			**13**			**9**	**100.0%**	**-3.53 [-8.34, 1.28]**
Heterogeneity: Not applicable								
Test for overall effect: Z = 1.44 (P = 0.15)								

Test for subgroup differences: Chi² = 0.26, df = 2 (P = 0.88), I² = 0%

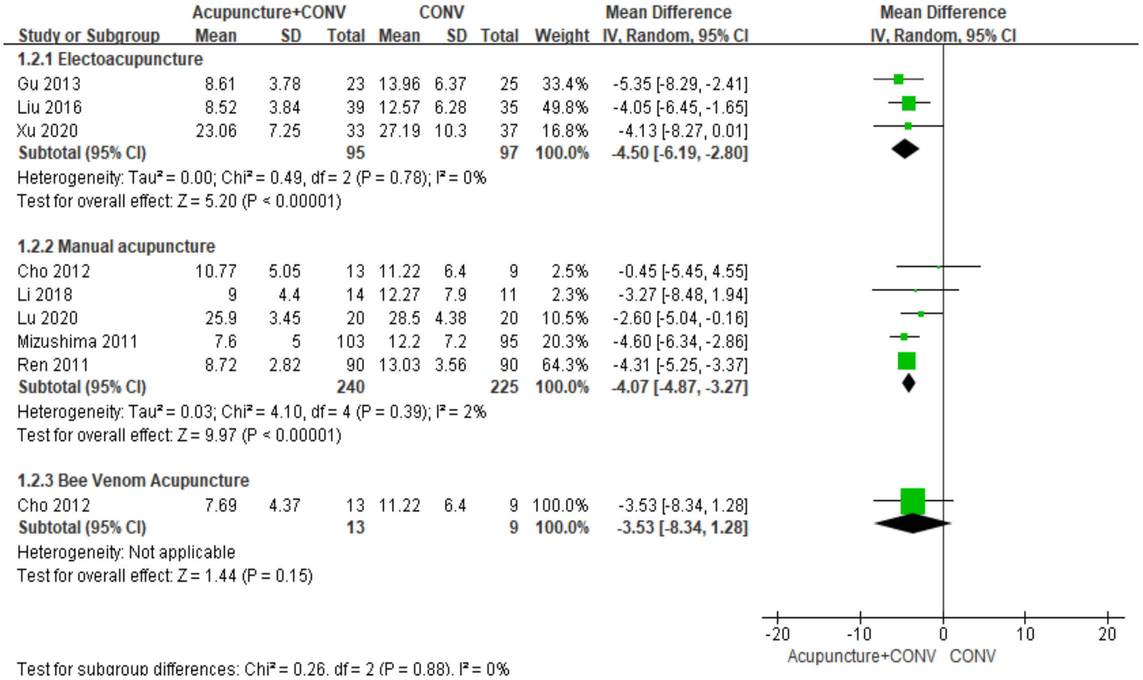

4.4 Acupuncture + Conventional drug therapy vs Sham Acupuncture + Conventional drug therapy, UPDRSII score

Study or Subgroup	Acupuncture+CONV Mean	SD	Total	SHAM+CONV Mean	SD	Total	Weight	Mean Difference IV, Random, 95% CI
**2.2.1 Electoacupuncture**								
Lei 2016	10.8	6.6	10	16.6	7.9	5	8.5%	-5.80 [-13.84, 2.24]
**Subtotal (95% CI)**			**10**			**5**	**8.5%**	**-5.80 [-13.84, 2.24]**
Heterogeneity: Not applicable								
Test for overall effect: Z = 1.41 (P = 0.16)								
**2.2.2 Manual Acupuncture**								
Jung 2006	8.88	5.737	16	8.57	6.867	21	33.3%	0.31 [-3.76, 4.38]
Li 2018	9	4.4	14	10.33	5.28	12	38.6%	-1.33 [-5.10, 2.44]
Park 2007	12.05	9.53	21	13.62	6.21	13	19.6%	-1.57 [-6.86, 3.72]
**Subtotal (95% CI)**			**51**			**46**	**91.5%**	**-0.79 [-3.24, 1.67]**
Heterogeneity: Tau² = 0.00; Chi² = 0.44, df = 2 (P = 0.80); I² = 0%								
Test for overall effect: Z = 0.63 (P = 0.53)								
**Total (95% CI)**			**61**			**51**	**100.0%**	**-1.21 [-3.56, 1.13]**
Heterogeneity: Tau² = 0.00; Chi² = 1.81, df = 3 (P = 0.61); I² = 0%								
Test for overall effect: Z = 1.01 (P = 0.31)								
Test for subgroup differences: Chi² = 1.37, df = 1 (P = 0.24), I² = 26.8%								

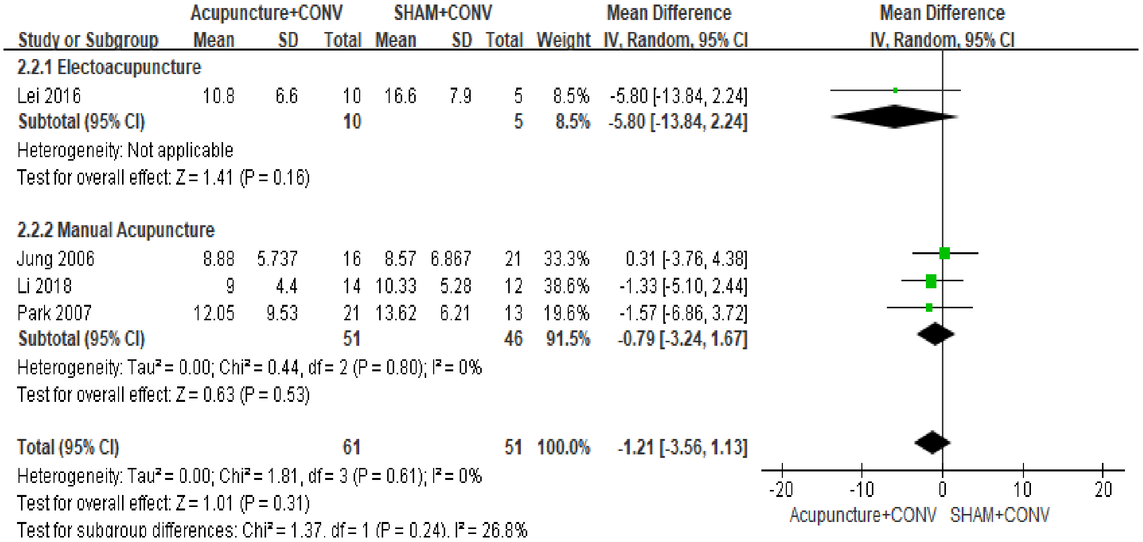

Supplement: Supplementary file 1 [file healthcare-09-01502-s001.zip › Supplement Digital Content 4_Forest Plot PMA.pdf]
