# Supplementary material for: Effect of Acupuncture on Movement Function in Patients with Parkinson’s Disease: Network Meta-Analysis of Randomized Controlled Trials
Source: Healthcare (Basel). 2021 Nov 5;9(11):1502. doi: 10.3390/healthcare9111502 (PMC8619200; doi:10.3390/healthcare9111502)
Supplement: Supplementary file 1 [file healthcare-09-01502-s001.zip › Supplement Digital Content 6_Sensitivity anal.pdf]

Supplemental Digital Contents. 6 Sensitivity analysis

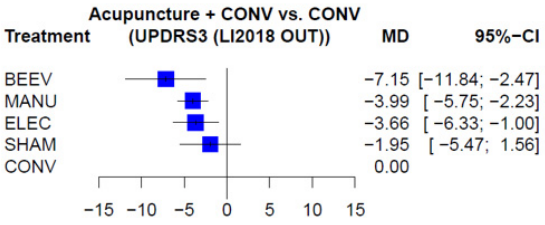

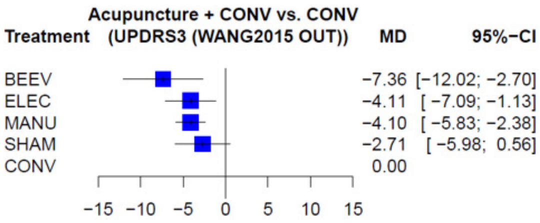

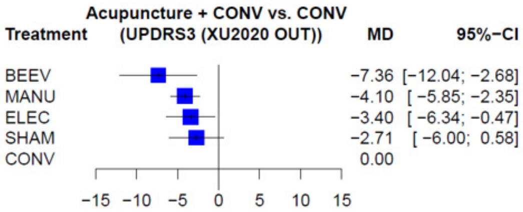

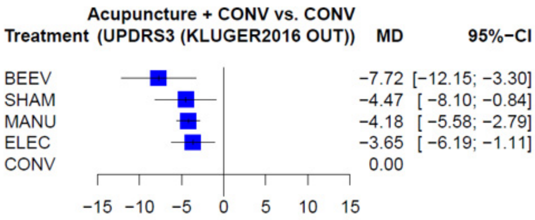

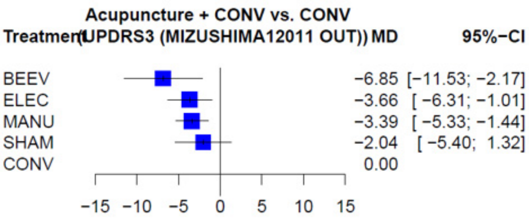

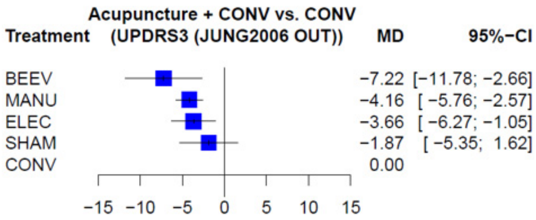

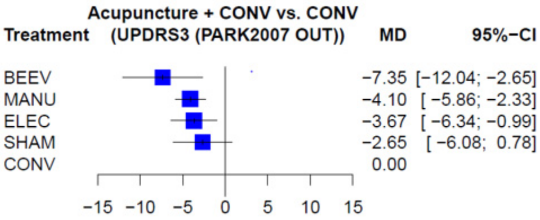

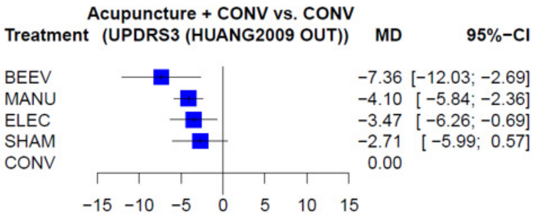

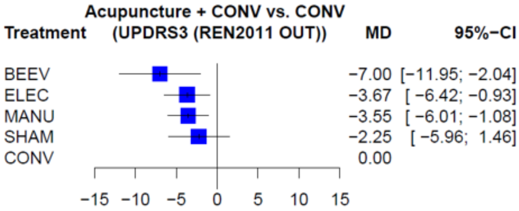

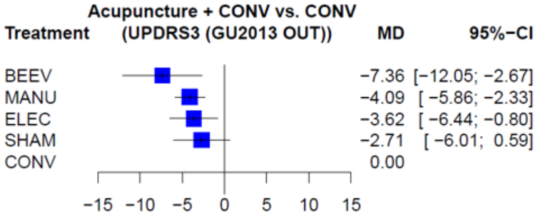

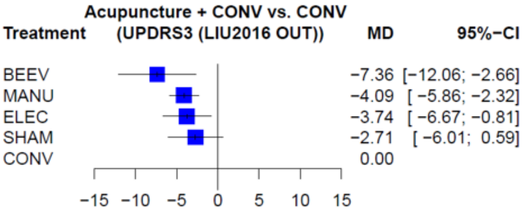

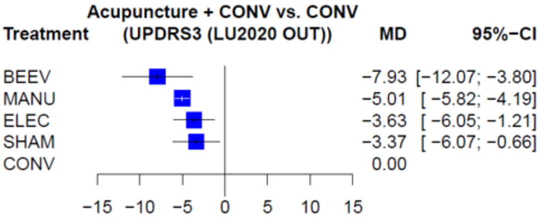

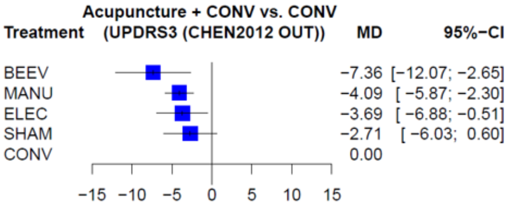

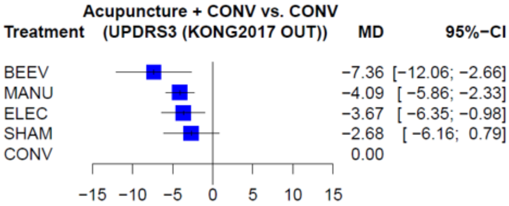

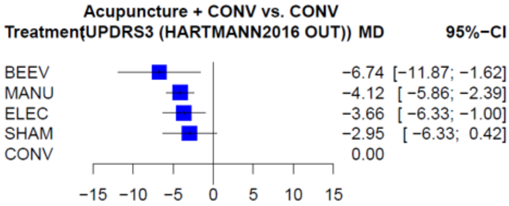

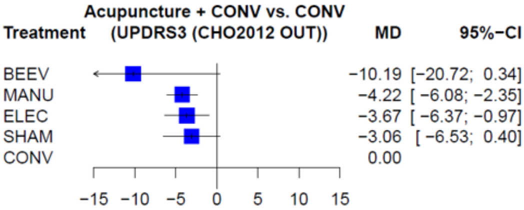

MD, Mean difference; CI, Confidence interval.

BEEV, Bee venom acupuncture+Conventional drug therapy; ELEC, Electroacupuncture+ Conventional drug therapy; SHAM, Sham acupuncture+Conventional drug therapy; CONV, Single conventional drug therapy; MANU, Manual acupuncture+Conventional drug therapy.
