# Supplementary material for: Effect of Acupuncture on Movement Function in Patients with Parkinson’s Disease: Network Meta-Analysis of Randomized Controlled Trials
Source: Healthcare (Basel). 2021 Nov 5;9(11):1502. doi: 10.3390/healthcare9111502 (PMC8619200; doi:10.3390/healthcare9111502)

Supplemental Digital Contents 7 Network plot, Forest plot, League table, Net split plot, and Funnel plot on secondary outcome (UPDRS II)

7.1 Network plot: UPDRSII score

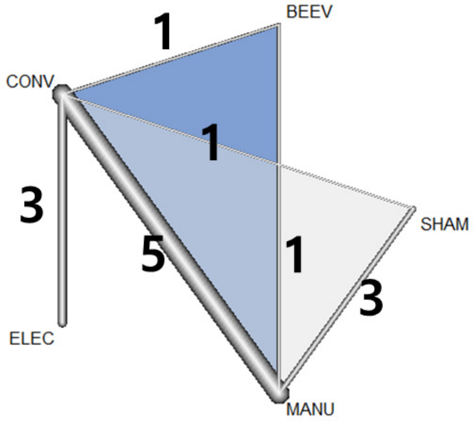

BEEV, Bee venom acupuncture + Conventional drug therapy; CONV, Single conventional drug therapy; ELEC, Electroacupuncture + Conventional drug therapy; MANU, Manual acupuncture + Conventional drug therapy; SHAM, Sham acupuncture + Conventional drug therapy

7.2 Treatment based forest plot : UPDRSII score

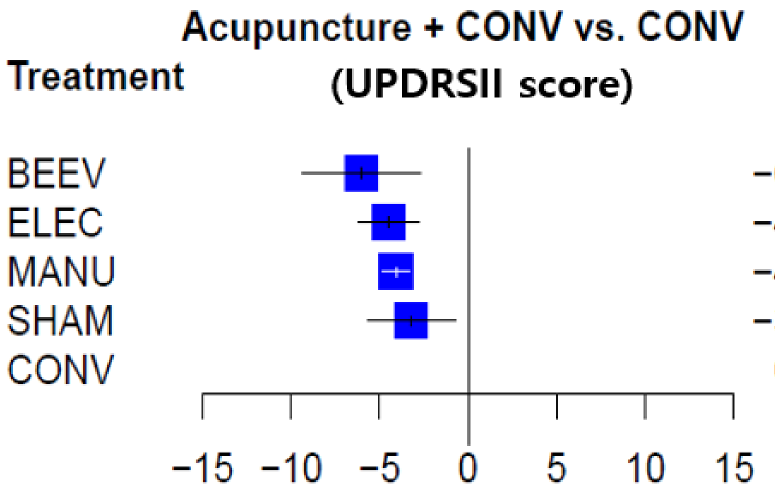

BEEV, Bee venom acupuncture + Conventional drug therapy; CONV, Single conventional drug therapy; ELEC, Electroacupuncture + Conventional drug therapy; MANU, Manual acupuncture + Conventional drug therapy; SHAM, Sham acupuncture + Conventional drug therapy; UPDRS, Unified Parkinson's disease rating scale.

7.3 League Table on UPDRS II

**BEEV**	-3.53(-8.34; 1.28)	.	-3.08(-6.71; 0.55)	.
**-6.07(-9.41; -2.72)**	**CONV**	**4.50(2.80; 6.19)**	**4.09(3.32; 4.85)**	1.94(-3.60; 7.48)
-1.57(-5.32; 2.18)	**4.50(2.80; 6.19)**	**ELEC**	.	.
-1.99(-5.30; 1.32)	**4.08(3.32; 4.84)**	-0.42(-2.28; 1.44)	**MANU**	-0.79(-3.24; 1.67)
-2.86(-6.95; 1.23)	**3.21(0.70; 5.72)**	-1.29(-4.32; 1.74)	-0.87(-3.29; 1.54)	**SHAM**

BEEV, Bee venom acupuncture + Conventional drug therapy; CONV, Single conventional drug therapy; ELEC, Electroacupuncture + Conventional drug therapy; MANU, Manual acupuncture + Conventional drug therapy; SHAM, Sham acupuncture + Conventional drug therapy; UPDRS, Unified Parkinson's disease rating scale.

The part highlighted in BOLD with underline is a comparison with statistically significant results. Upper right triangle present effect size estimated by only direct comparison. As direct comparison does not exist in all treatment comparison, there are several blank in upper right triangle. Lower left triangle provide pooled estimation of direct and indirect comparison of effect size.

7-4 Network net split plot: UPDRS II score

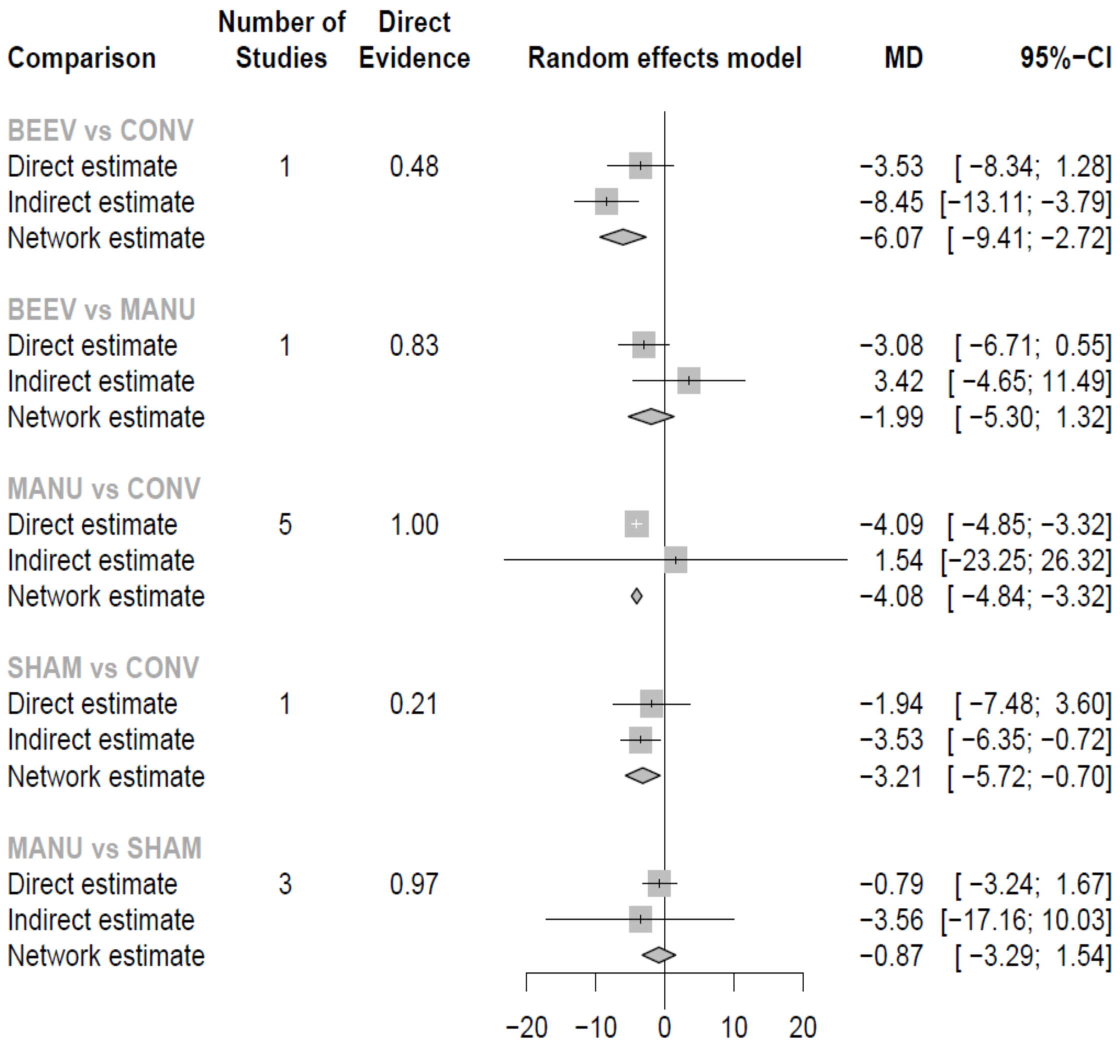

7.5 Funnel plot : UPDRS II

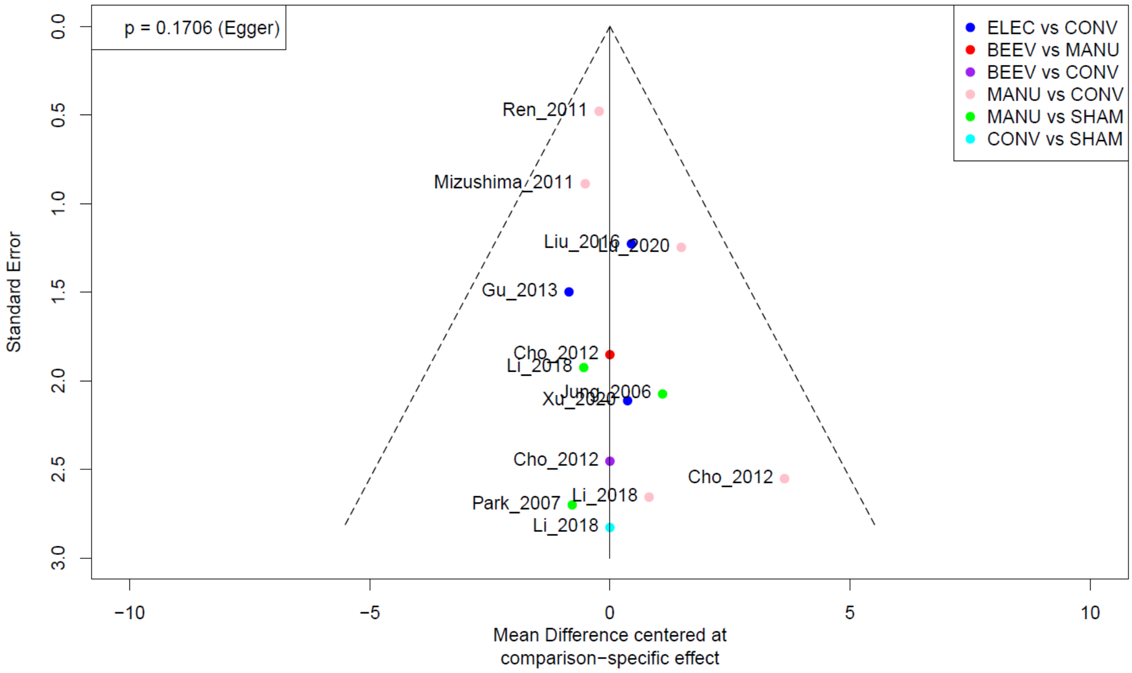

Supplement: Supplementary file 1 [file healthcare-09-01502-s001.zip › Supplement Digital Content 7_UPDRS2.pdf]
