# Supplementary figures and images for: Effect of Acupuncture on Movement Function in Patients with Parkinson’s Disease: Network Meta-Analysis of Randomized Controlled Trials
Source: Healthcare (Basel). 2021 Nov 5;9(11):1502. doi: 10.3390/healthcare9111502 (PMC8619200; doi:10.3390/healthcare9111502)

# Supplemental Digital Content 5 Net-split plot: UPDRS III score

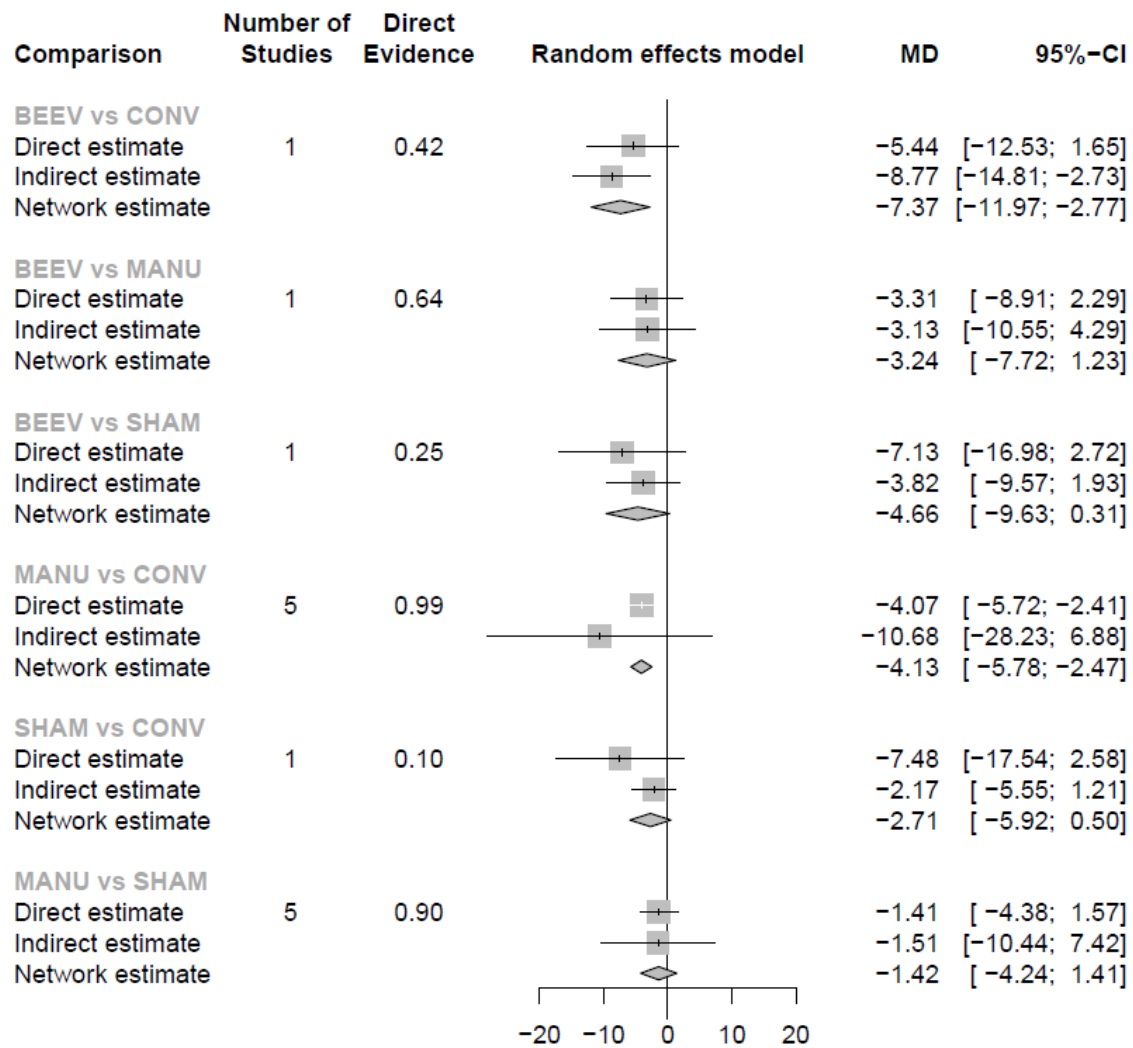

Supplement: Supplementary file 1 [file healthcare-09-01502-s001.zip › Supplement Digital Content 5_Net split graph.pdf]
